# Stage-stratified benefits of AI-radiomics PET in early Alzheimer’s disease: a systematic review and meta-analysis

**DOI:** 10.3389/fneur.2026.1771993

**Published:** 2026-05-15

**Authors:** Jinglu Duan, Meixuan Yang, Yong Wang

**Affiliations:** 1 Medical Imaging Department, Hebei Medical University, Shijiazhuang, China; 2 Department of Radiology and Nuclear Medicine, The First Hospital of Hebei Medical University, Shijiazhuang, China

**Keywords:** Alzheimer’s disease, artificial intelligence, meta-analysis, positron emission computed tomography, radiomics

## Abstract

**Objective:**

AI-radiomics can analyze radiological images more thoroughly and quickly than the human eye. This study aims to compare the diagnostic efficacy of AI-assisted PET radiomics for Alzheimer’s disease (AD) with conventional PET diagnosis through a systematic review and bivariate meta-analysis performing indirect, study-level benchmarking versus conventional PET.

**Methods:**

PubMed, Embase, and Web of Science were searched through April 11, 2025, for human diagnostic accuracy studies for AI-assisted PET radiomics. Two reviewers extracted data per PRISMA guidelines, risk and bias were appraised using QUADAS-AI. Effect sizes were synthesized via a bivariate random-effects model with HSROC. Prespecified strata contrasted with AD vs. healthy controls (HC), AD vs. mild cognitive impairment (MCI), and tracer class. The analyses were conducted based on bivariate random-effects model realized using R and Stata.

**Results:**

Nine studies (25 2 × 2 tables; *n* = 5,765) were included. A strong correlation between sensitivity and specificity signaled substantial study heterogeneity. This heterogeneity was further illustrated by the dispersion of the HSROC prediction region. In AD vs. HC, proteinopathy PET yielded SE 0.89, SP 0.91, and AUC 0.96. In comparison, the ^18^F-FDG PET demonstrated near-parity (SE 0.92, SP 0.92 AUC 0.94), suggesting limited incremental value. In AD vs. MCI, current data suggested a trend toward improved performance with proteinopathy PET relative to ^18^F-FDG PET (SE 0.94, SP 0.95, AUC 0.96 vs. AUC 0.84). These results underscore the potential of proteinopathy PET in facilitating early diagnostic evaluations, necessitating further validation. In contrast to conventional benchmarks, the AD vs. MCI demonstrated notably higher diagnostic metrics (AUC 0.96; LR + 19.64; LR − 0.06; conventional amyloid-PET specificity approximately 0.49), while the gains in AD vs. HC were negligible (ΔAUC +0.02). Sensitivity analyses confirmed that primary estimates were not influenced by a single study.

**Conclusion:**

AI-radiomics on proteinopathy PET shows promising potential for distinguishing AD from MCI, yet only marginal benefits comparing AD to HC. However, given the heterogeneity of the data, the risk of bias, and the limited external validation, there is a need to prioritize multi-site validation, standardized reporting, and prospective decision-impact studies.

**Systematic review registration:**

https://www.crd.york.ac.uk/PROSPERO/view/CRD420251029823, identifier, PROSPERO (CRD420251029823).

## 1 Introduction

Alzheimer’s disease is the leading cause of dementia worldwide (1), accounting for 60–80% of cases and imposing substantial pressure on public health systems in aging societies (2). Conventional diagnostic pathways such as clinical assessment, neuropsychological testing, and visual/quantitative reads of CT, MRI, and PET scans that can underperform in early and atypical presentations (3). Within the A/T/N framework, amyloid- PET, tau-PET and FDG-PET capture pathology and neurodegeneration that often precede symptoms by years, underscoring the need for more sensitive analytic approaches (4). AI-assisted radiomics may unlock signal beyond standard visual interpretation by leveraging high-dimensional texture, morphology, and intensity features from PET.

Positron emission tomography is a highly advanced radiographic technique that utilizes tracers, such as ^18^F-fluoro-2-deoxyglucose (^18^F-FDG), for the assessment of cerebral metabolism (5). Pathological protein tracers, including ^18^F-Florbetapir (6) are used for the identification of early neurodegenerative alterations. Nevertheless, current clinical practice primarily relies on clinical visual evaluation and standardized uptake value ratios (SUVRs) (7). This method potentially fails to recognize the intricacy and nuance characteristic of diseases (8).

The development of more sensitive diagnostic tools for early-stage and atypical Alzheimer’s disease has created a need for innovative analytical methodologies that can detect subtle changes in PET imaging data. At the present time, radiomics represents the optimal solution. Radiomics involves the systematic extraction and analysis of a multitude of quantitative imaging features, including textural, morphological, and intensity-based parameters, from PET scans. The purpose of radiomics is to facilitate the identification of disease-specific patterns that are imperceptible through conventional visual interpretation (9). Additionally, the utilization of artificial intelligence (AI), particularly machine learning (ML) and deep learning (DL) algorithms, has been applied in the field of feature recognition in PET diagnosis, including both visual evaluation and standardized uptake value ratios (SUVs). This assertion is supported by the findings of prior studies (10). Moreover, when employed in the context of radiomics diagnosis, it serves as a more powerful too. Such enhanced approaches have been demonstrated to process and evaluate data at a considerably faster rate than previous methods while also ensuring greater precision (11). Research in the field of radiomics has consistently demonstrated superior diagnostic performance in comparison with conventional imaging modalities. This finding underscores the promise of radiomics in facilitating the early diagnosis of Alzheimer’s disease (12).

Addressing a critical evidence gap left by prior broad reviews of AI in neuroimaging, and PET for Alzheimer’s disease, a PRISMA-DTA conformant systematic review and bivariate meta-analysis was conducted, with a focus on AI-assisted PET radiomics. The estimation of pooled sensitivity, specificity, and HSROC AUC was stratified by clinical contrast (AD vs. HC; AD vs. MCI), and benchmarked against conventional PET performance reported in recent meta-analyses. Furthermore, the study-level variability was quantified (95% HSROC prediction regions), the moderators were probed (DL vs. ML; sample size), and the risk of bias was characterized using QUADAS-AI. Collectively, these analyses deliver stage-stratified performance estimates to inform clinical adoption and mitigate future research roadmaps.

## 2 Methods

This meta-analysis followed PRISMA-DTA (13) and PRISMA 2020 (14) standards.

### 2.1 Search strategy

A systematic literature search was performed using PubMed, Embase, Web of Science databases to identify studies published from inception to April 11, 2025. The following Medical Subject Headings (MeSH) and keyword terms were used in combination: (Alzheimer disease) AND (radiomics) AND (artificial intelligence OR machine learning OR deep learning). Search terms within each thematic group were combined using the Boolean operator “OR” and the thematic groups were then combined using the Boolean operator “AND.” Comprehensive strategies for each database are provided in the Supplementary material. Furthermore, the reference lists of relevant articles were manually screened to identify any additional eligible studies. The literature search and screening were independently performed by two authors (JLD and MXY). Disagreements were resolved through discussion.

### 2.2 Inclusion and exclusion criteria

Eligible articles were selected based on strict adherence to the PICOS framework (Population, Intervention, Comparison, Outcome, Study design). Inclusion criteria were as follows: (1) population: individuals with a confirmed diagnosis of AD, MCI and healthy controls (individuals with no significant clinical symptoms related to AD); (2) intervention: neurological diagnosis made based on positron emission tomography radiomics with AI method; (3) comparison: conventional PET visual read or SUVR threshold. Reference standard: histopathology or ≥12-month follow-up with clinical consensus diagnosis; (4) outcome: Primary: sensitivity, specificity, AUC (AD vs. HC; AD vs. MCI). Secondary: LR+, LR−, DOR, threshold effects, calibration/heterogeneity; and (5) Cross-sectional or cohort; prospective preferred; multi-center; n ≥ 30; STARD-aligned reporting; blinded, independent interpretation. Exclusion criteria included: (1) non-AI, (2) non-PET, (3) reviews, (4) case reports, and (5) non-English.

### 2.3 Comparator evidence harvesting and alignment

We conducted a comprehensive search of PubMed and PMC over the past 5 years to identify human meta-analyses of PET in early Alzheimer’s disease. These analyses reported sensitivity, specificity, and, where available, AUC. The studies were stratified by AD vs. HC and AD vs. MCI. Eligible modalities included ^18^F-FDG, amyloid PET, and tau PET. From each meta-analysis, we abstracted pooled point estimates (95% CIs) and derived LR+, LR−, and DOR for benchmarking.

### 2.4 Data extraction

The characteristics and diagnostic performance were extracted independently by two reviewers using a standardized data extraction sheet. Discrepancies were resolved by consensus. Information was collected in a data set, including participants’ demographics, inclusion and exclusion criteria, the total number of subjects included in the study, a reference standard, data characteristics, imaging agents, information about poor image quality, data sources, details regarding the design of the algorithms, the types of algorithms, and the methods of validation. In addition, data regarding diagnostic accuracy was collected.

The binary diagnostic accuracy data were extracted, and contingency tables were constructed at the reported thresholds. The diagnostic accuracy data, which included measures such as sensitivity (SE), specificity (SP), area under the curve (AUC), true-positive (TP), false-positive (FP), true-negative (TN), and false-negative (FN) values, were extracted directly into contingency tables for the AI model. These data were then utilized to calculate SE and SP. If a study compared different models or algorithms, only the main research subject will be extracted. The contingency tables for the included studies are summarized in Supplementary Table 10. For the primary analysis, all available relevant data splits (e.g., training, internal validation, and external test sets) to capture overall performance trends. Conversely, for the sensitivity analysis addressing within-study dependence, we restricted the data to a single prespecified 2 × 2 table per study and tracer-comparison according to a protocolized hierarchy: (1) the operating point evaluated on an external validation set; if unavailable, (2) the author-declared primary clinical threshold; if unavailable, (3) the prespecified threshold used for the main analysis in the paper.

### 2.5 Quality assessment

The risk of bias and applicability of all selected studies were assessed by using the QUADAS-AI criteria (15). It furnishes researchers with a particular framework for evaluating the risk of bias and applicability when conducting reviews that evaluate the accuracy of AI-assisted diagnostic tests. Furthermore, an applicability analysis was conducted. A thorough review and analysis of all studies was conducted by at least two independent authors. Disagreements were resolved through consensus.

### 2.6 Statistical analysis

The objective of the present study was to estimate the diagnostic performance of AI-assisted radiomics in PET and compare it with the performance of the current method. To this end, a meta-analysis of studies with contingency tables was conducted. The random-effects model was employed due to the presumed disparities among the studies. We fit a bivariate random-effects (Reitsma) model to obtain pooled sensitivity and specificity and to plot HSROC curves with 95% confidence and prediction regions. Between-study variability is presented via the HSROC prediction region; univariate I^2^ was not used for DTA synthesis (16). The combined curve was plotted with the corresponding 95% confidence region and 95% prediction region around the averaged estimates of SE, SP, and AUC in the HSROC figures. Small-study effects were assessed using Deeks’ funnel-plot asymmetry test (*p*-value reported for each analysis). Prespecified subgroup meta-regressions included algorithm class (DL vs. ML) and sample size (≥100 vs. < 100).

Considering the observed discrepancy in clinical utility between the control group and the other study groups, the included studies were initially separated into two classifications: AD from HC group and AD from MCI group. The diagnostic performance of the different tracers (glucose metabolism, pathological protein) was evaluated separately, as the functional and regional brain uptakes vary in the radioligands. Subsequently, to identify the source/sources of extreme heterogeneity, a subgroup analysis was conducted based on the following criteria: (1) AI algorithms (ML or DL), and (2) the sample size of the AI algorithms (≥100 or <100). Comparative effects versus conventional PET were summarized as ΔAUC and Δlogit-Se/Δlogit-Sp from bivariate random-effects models; LR+/LR−/DOR and 95% prediction intervals were computed for decision relevance. To investigate the sources of heterogeneity among studies, a meta-regression analysis was conducted, with the type of AI algorithms and sample size considered as covariates.

Analyses were conducted in R 4.5.1 (R Foundation; RRID: SCR_001905) and Stata version18 (StataCorp LP, College Station, TX, United States; MIDAS and MetaDTA; RRID: SCR_012763). We prespecified *α* = 0.05 (two-sided), report exact *p*-values, and present very small values as *p* < 0.001.

## 3 Results

### 3.1 Literature screening results

**Figure 1:**
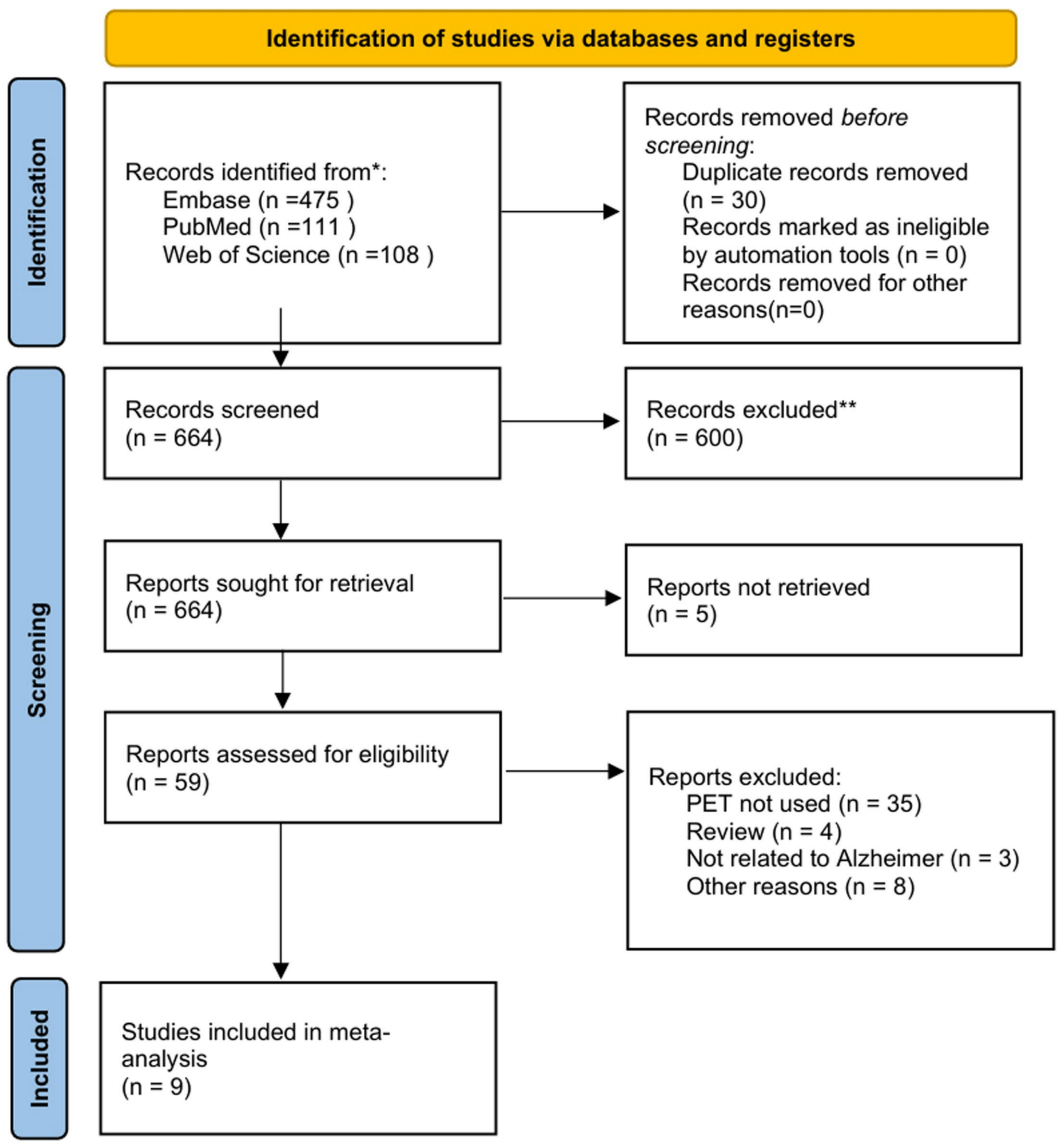
PRISMA flowchart of study selection.

The preliminary search yielded a total of 694 records, of which 30 were duplicates. Following the screening of titles and abstracts, 600 studies were excluded, leaving 64 articles for full-text eligibility assessment. Following a thorough review of the literature, five studies were deemed to be of insufficient quality to be included in the analysis. This resulted in a total of 59 studies being selected for qualitative synthesis. However, 50 of these studies were excluded for various reasons. After a further literature review, a total of 9 articles were found to contain sufficient data to meet the inclusion criteria for meta-analysis. The literature screening process is shown in Figure 1.

### 3.2 Characteristics of the included studies

The detailed characteristics of these included studies are shown in Table 1 and Supplementary Table 1. It is notable that all studies utilize retrospective data. Seven studies utilized images from public databases. All studies recruited patients based on routine clinical diagnosis. Regarding the imaging techniques employed, two studies utilized both PET and MRI to train the AI model, while the remaining seven relied exclusively on PET imaging. Two studies utilized out-of-sample datasets for the purpose of external validation. “All studies used a single PET tracer category: proteinopathy PET (amyloid or tau) or ^18^F-FDG. Moreover, the distribution of studies concerning the classification of AD in the present study is as follows: A total of six studies were conducted on the classification of AD from MCI, and five studies were conducted on the classification of AD from HC (more details see Supplementary Table 2). Supplementary Tables 3, 4 provide a detailed enumeration of the various categories using different PET imaging tracers. Tables 2, 3 summarize the estimate of the pooled performance of AI–assisted PET imaging for the diagnosis of AD. Forest plots can be found in the Supplementary Figures 1–6.

**Table 1:** Characteristics of all included studies (*n* = 9).

Author year(ref)	Algorithm details							Data characteristics				
	Model	Number of patients	Training/ validation(ratio)	Testing	Type of internal validation	External validation	ML/DL	Target condition	Imaging agent	Source of data	Prospective	Data range
Jiang et al., 2024 (54)	CNN; XGBoost	1962	NR	NR	NR	Yes	DL + ML	AD vs. MCI	^18^F-FDG	ADNI public database; Huashan Hospital, Shanghai & Xuanwu Hospital, Beijing	No	NR
Mu et al., 2024 (55)	GBDT; LR	278	222 (4:1)	56	Single hold-out validation set (no k-fold CV)	No	ML	AD vs. MCI	^18^F-FDG	ADNI-2 public database	No	NR
Chen et al., 2024 (56)	mRMR; LR	159	113(7:3)	46	5-fold cross-validation	No	ML	AD vs. HC AD vs. MCI	^18^F-FDG	Dept. Radiology & Nuclear Med., Xuanwu Hospital, Capital Medical Univ., Beijing	No	Jul 2017 – Aug 2022
Peng et al., 2023 (57)	mRMR; GBDT; SVM; NB; RF; KNN	341	238	103	10-fold cross-validation	No	ML	AD vs. MCI	^18^F-FDG	ADNI public database	No	NR
Alongi et al., 2022 (58)	DA	43	NR	NR	5-fold cross-validation	No	ML	AD vs. HC	^18^F-FDG	G. Giglio Institute, Cefalù (Italy)	No	Jul 2016 – Sep 2017
Zhao et al., 2022 (48)	ResNet34; SVM	513	463	50	10-fold cross-validation (200 times)	No	DL	AD vs. MCI vs. NC	^18^F-Flortaucipir	ADNI public database	No	NR
Ding et al., 2021 (59)	SVM	619	334	285	10-fold cross-validation (1,000 times)	Yes	ML	AD vs. HC	^18^F-Florbetapir	ADNI public database	No	NR
Zhou et al., 2021 (60)	ZF-Net; SVM	355	321	34	10-fold cross-validation (100 times)	No	DL	AD vs. MCI	^18^F-FDG	ADNI public database	No	NR
Dai et al., 2021 (61)	DeU-Net	182	NR	NR	10-fold cross-validation	No	DL	AD vs. HC	^18^F-FDG	ADNI public database & PPMI public database	No	NR

**Table 2:** Summary estimates and meta-regression of pooled performance of AI-assisted PET imaging in the diagnosing AD from HC.

Parameter	No. of tables	AUC (HSROC 95% CI)	SE (95% CI)	*p* value^a^	SP (95% CI)	*p* value^a^	LR + (95% CI)	LR- (95% CI)
Proteinopathy Overall	5	0.96 (0.86–0.98)	89 (73–96)		91 (86–98)		6.41 (3.79–10.84)	0.17 (0.10–0.30)
^18^F-FDG Overall	5	0.94 (0.93–0.97)	92 (89–94)		92 (86–96)		12.03 (6.58–22.00)	0.09 (0.07–0.11)
Algorithm				<0.001		<0.001		
DL	5	0.96 (0.92–0.99)	93 (90–96)		95 (85–99)		14.52 (6.87–30.68)	0.08 (0.05–0.12)
ML	5	0.91 (0.82–0.94)	83 (74–89)		87 (82–91)		5.96 (3.82–9.29)	0.20 (0.13–0.33)
Sample size				<0.001		<0.001		
≥100	7	0.95 (0.90–0.98)	89 (80–94)		90 (85–93)		8.13 (5.06–13.05)	0.13 (0.08–0.22)
<100	3	0.97 (0.93–0.99)	90 (80–96)		95 (86–98)		24.61 (7.31–82.91)	0.09 (0.04–0.21)

HC, healthy controls; AD, Alzheimer’s disease; DL, deep learning; ML, machine learning; ^18^F-FDG, ^18^F-fluorodeoxyglucose; proteinopathy PET: amyloid or tau; LR+, positive likelihood ratio; LR−, negative likelihood ratio; SE, sensitivity; SP, specificity; AUC, area under the HSROC curve.

a *p* value for heterogeneity between subgroups with meta-regression analysis.

**Table 3:** Summary estimates and meta-regression of pooled performance of AI-assisted PET imaging in the diagnosing AD from MCI.

Parameter	No. of tables	AUC (HSROC95% CI)	SE (95% CI)	*p* value^a^	SP (95% CI)	*p* value^a^	LR + (95% CI)	LR-(95% CI)
Proteinopathy overall	2	0.96 (0.92–0.98)	94 (85–98)		95 (80–99)		19.64 (4.09–94.30)	0.06 (0.03–0.12)
^18^F-FDG overall	13	0.84 (0.81–0.88)	82 (79–85)		74 (67–80)		3.25 (2.60–4.07)	0.22 (0.18–0.27)
Algorithm				<0.001		<0.001		
DL	5	0.94 (0.87–0.97)	89 (82–93)		91 (80–96)		10.46 (3.71–29.46)	0.14 (0.08–0.24)
ML	10	0.84 (0.77–0.88)	82 (76–86)		71 (63–77)		2.94 (2.31–3.76)	0.24 (0.17–0.32)
Sample size				<0.001		<0.001		
≥100	6	0.91 (0.84–0.95)	87 (81–91)		85 (71–93)		5.02 (3.40–7.40)	0.15 (0.10–0.23)
<100	9	0.82 (0.78–0.89)	79 (62–82)		73 (62–82)		2.98 (2.06–4.31)	0.27 (0.19–0.38)

a *p* value for heterogeneity between subgroups with meta-regression analysis.

### 3.3 Study quality

The risk of bias was evaluated using QUADAS-AI (see Supplementary Figure 18; Supplementary Table 9, as well as Supplementary Table 11 for the full signaling items). Across the nine studies, the risk of bias was uniformly low for patient selection, reference standard, and flow/timing (9/9, 100% each). The index-test domain concentrated on the issues: Seven out of nine (77.8%) were rated high risk, driven by a lack of external validation (Q6 “No” in seven out of nine), with only two out of nine (22.2%) deploying an independent test set. Overall, bias is largely contained outside the index-test domain; however, generalizability is materially constrained by internal-only validation. Future studies should include external cohorts (pre-registered splits), publish detailed data partitioning, and report model performance on fully held-out datasets to reduce optimism and strengthen applicability.

### 3.4 Meta-analysis results

#### 3.4.1 Pooled performance of AI algorithms for classifying AD from HC

**Figure 2:**
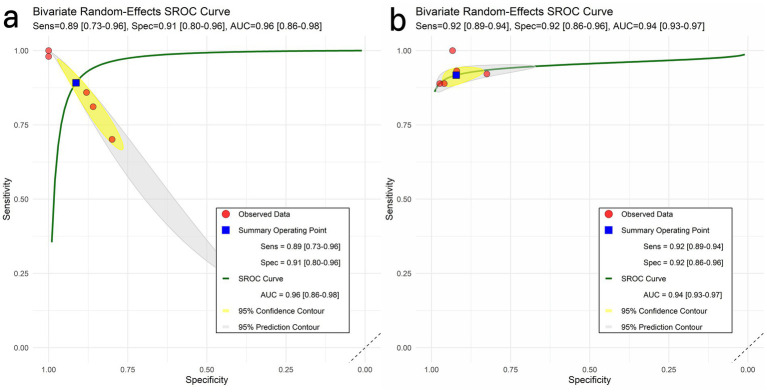
HSROC curves for classifying AD vs HC. Panels: **(a)** pathology-PET; **(b)** 18F-FDG PET. Shaded areas denote 95% prediction regions (between-study heterogeneity).

A total of five contingency tables from two studies are employing proteinopathy PET imaging. The pooled sensitivity (SE), specificity (SP), and area under curve (AUC) for this group were 89% (95% CI: 73–96), 91% (95% CI: 80–96), and 0.96 (95% CI: 0.86–0.98), respectively (Figure 2).

Three studies utilizing ^18^F–FDG PET imaging yielded sufficient data for the construction of contingency tables and the calculation of diagnostic performance metrics. For the present studies, the pooled SE, SP, and AUC were 92% (95% CI: 89–94), 92% (95% CI: 86–96), and 0.94 (95% CI: 0.93–0.97), respectively.

Concerning AI algorithms, the hierarchical summary receiver operating characteristic (HSROC) curves for the aforementioned algorithms are illustrated in Supplementary Figure 8. The pooled SE for DL was 93% (95% CI: 90–96), and for ML was 83% (95% CI: 74–89); pooled SP was 95% (95% CI: 85–99) for DL and 87% (95% CI: 82–91) for ML. The AUC was 0.96 (95% CI: 0.92–0.99) for DL and 0.91 (95% CI: 0.82–0.94) for ML.

With respect to sample sizes, we determined that 100 patients is a critical criterion for this analysis. The hierarchical HSROC curves for these sample size subgroups are shown in Supplementary Figure 9. The pooled SE for samples larger than 100 was 89% (95% CI: 80–94), and for samples smaller than 100 was 90% (95% CI: 80–96); pooled SP was 90% (95% CI: 85–93) for larger samples and 95% (95% CI: 86–98) for smaller samples. The AUC was 0.95 (95% CI: 0.90–0.98) for the larger sample size group and 0.97 (95% CI: 0.93–0.99) for the smaller one.

#### 3.4.2 Pooled performance of AI algorithms for classifying AD from MCI

**Figure 3:**
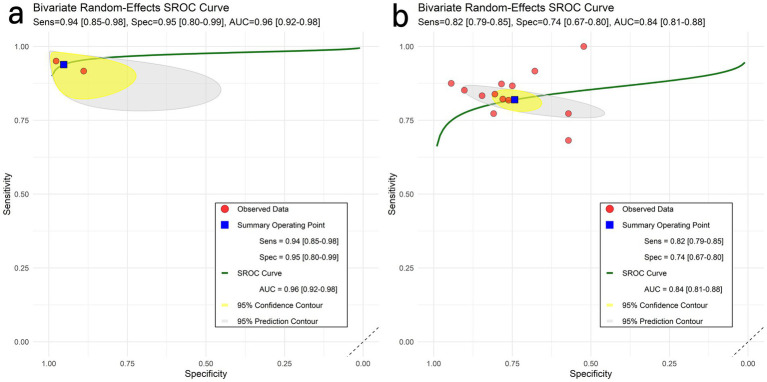
HSROC curves for classifying AD vs MCI. Panels: **(a)** pathology-PET; **(b)** 18F-FDG PET. Shaded areas denote 95% prediction regions.

Contingency tables derived from proteinopathy PET imaging analyses have been incorporated. The pooled SE, SP, and AUC were 94% (95% CI: 85–98%), 95% (95% CI: 80–99%), and 0.96 (95% CI: 0.92–0.98), respectively (Figure 3).

For the ^18^F–FDG PET imaging study, a total of 13 contingency tables were incorporated into the analysis. The pooled results within this group demonstrated an SE of 82% (95% CI: 79–85), a SP of 74% (95% CI: 67–80), and an AUC of 0.84 (95% CI: 0.81–0.88).

Concerning the matter of AI algorithms (ML/DL), the respective HSROC curves for these algorithms are illustrated in Supplementary Figure 11. The pooled SE for DL was 89% (95% CI: 82–93), and for ML was 82% (95% CI: 76–86); pooled SP was 91% (95% CI: 80–96) for DL and 71% (95% CI: 63–77) for ML. The AUC was determined to be 0.94 (95% CI: 0.87–0.97) for DL and 0.84 (95% CI: 0.77–0.88) for ML.

In terms of sample sizes, with criterion of 100 patients, the HSROC curve is illustrated in Supplementary Figure 12. The pooled SE for groups with over 100 samples was 87% (95% CI: 81–91), whereas for groups with fewer than 100 samples it was 79% (95% CI: 62–82). The pooled SP for the larger sample group was 85% (95% CI: 71–93), and for those with fewer than 100 samples, it was 73% (95% CI: 62–82). The AUC was 0.91 (95% CI: 0.84–0.95) for larger samples and 0.82 (95% CI: 0.78–0.89) for smaller samples.

#### 3.4.3 Sensitivity analysis

Leave-one-out sensitivity analyses for both AD vs. HC and AD vs. MCI comparisons showed that summary sensitivity and specificity remained stable across most exclusion scenarios, indicating that the primary diagnostic accuracy estimates were not driven by any single study. In several subsets, boundary solutions or model instability were observed, reflecting limited information to support the full HSROC structure rather than changes in diagnostic performance.

Furthermore, to address potential within-study dependence, we conducted a rigorous sensitivity analysis restricting the dataset to a single prespecified estimate per independent study. For the AD vs. MCI (18F-FDG) subgroup (*N* = 6), the independent pooled AUC remained robust at 0.90. For other subgroups with restricted sample sizes (N < 4), we performed descriptive sensitivity checks. The independent AUCs ranged from 0.928 to 0.963, closely mirroring our primary pooled estimates. Due to the small sample sizes, these findings serve as descriptive sensitivity checks rather than confirmatory evidence, indicating that multiple extractions are unlikely to have materially biased our primary clinical findings.

#### 3.4.4 Heterogeneity analysis

Between-study variability was non-trivial across contrasts, as indicated by wide HSROC prediction regions. In meta-regressions, algorithm class (DL vs. ML) and sample size (≥100 vs. < 100) partially explained dispersion (*p*-values in Tables 2, 3); subgroup curves are shown in Supplementary Figures. Deeks’ funnel plots assessed small-study effects (Supplementary Figures 13–17).

Detailed results of all subgroups and meta-regression analyses examining the potential source of heterogeneity between studies are shown in Tables 2, 3 and Supplementary Tables 7–10. The results indicate statistically significant differences in the covariates.

#### 3.4.5 External benchmarking

In AD vs. HC, AI-radiomics proteinopathy PET achieved SE 0.89 / SP 0.91 / AUC 0.96, versus a conventional amyloid-PET benchmark of SE 0.91 / SP 0.81 / AUC 0.91 (17); the incremental gain over AI assisted ^18^F-FDG radionics was nominal (AUC 0.96 vs. 0.94; ΔAUC +0.02). In AD vs. MCI, AI-radiomics proteinopathy PET delivered SE 0.94 / SP 0.95 / AUC 0.96 (LR + 19.64; LR − 0.06), contrasting with conventional amyloid-PET’s low specificity (~0.49) and with AI assisted ^18^F-FDG radionics (AUC 0.84) (17).

## 4 Discussion

The application of AI-radiomics on proteinopathy PET was associated with higher diagnostic performance, particularly at the prodromal stage. In the distinction between AD and MCI, the performance metrics demonstrate significant efficacy, with an AUC of 0.96, an LR + of 19.64, and an LR- of 0.06. This observation not only addresses the conventional limitations of amyloid-PET in terms of specificity but also yielded higher diagnostic metrics compared to AI assisted ^18^F-FDG radiomics. These preliminary findings suggest that AI-radiomics could potentially serve as a supplementary tool (e.g., a concurrent reader) in memory-clinic pathways, pending prospective validation. In scenarios where proteinopathy PET is unavailable, the study suggests the adoption of AI-FDG, which exhibits diagnostic accuracy comparable to that of AD vs. HC.

AI-radiomics proteinopathy PET provided high diagnostic accuracy in MCI, with an AUC of 0.96, LR + of 19.64, and LR − of 0.06 (SE 0.94; SP 0.95). In contrast, AI-assisted ^18^F-FDG radionics showed notably lower performance (AUC 0.84; LR + 3.25; LR − 0.22). In the comparison of AD vs. HC, both modalities were near ceiling performance (AUC 0.96 vs. 0.94; ΔAUC +0.02), a difference unlikely to change management. Therefore, MCI workups represent highly promising clinical application for proteinopathy PET with AI.

Benchmarking against conventional amyloid-PET further underscores this gradient: specificity is approximately 0.49 for AD vs. MCI under conventional reads, compared to 0.95 with AI-radiomics proteinopathy PET. Meanwhile, the differences between AD vs. HC are modest relative to high baselines.

It is important to note that AI models which achieve a high level of accuracy in research cohorts may not necessarily perform equally well in underrepresented populations. Prior to clinical deployment, validation in populations representative of intended use settings is essential.

Model instability or boundary solutions observed in some sensitivity analyses reflect the limited number of studies and high correlation between sensitivity and specificity, indicating structural dependence of the HSROC model rather than changes in diagnostic accuracy. The consistency of summary estimates supports the robustness of the primary findings.

In this study, we incorporated not only conventional machine learning techniques but also recent publications on deep learning, a methodology that has witnessed a surge in popularity in recent years. The “black-box” nature of DL can hinder clinician trust; several studies have reported post-hoc explanations that localize AD-concordant features, thereby improving auditability (18–21). Given the observation that DL models tended to exhibit higher pooled accuracy than ML models in our dataset, this raises a pivotal translational challenge: how to utilize superior accuracy in clinical decision-making while managing reduced interpretability and clinician trust. Performance enhancement in isolation is inadequate for deployment in high-stakes settings. It is imperative that clinicians possess the ability to interrogate or, at the very least, conceptualize the rationale underpinning the model’s capacity to support a specific classification. Post-hoc explanation methods that highlight AD-concordant patterns provide a practical bridge between DL internal representations and established disease knowledge, rendering outputs more audit-ready. However, it must be noted that these explanations can themselves be unstable or misleading. Therefore, it is essential that their use is standardized and embedded in clear operating procedures to avoid becoming an additional source of opacity.

A complementary strategy is to differentiate the roles of DL and ML within the clinical workflow. The application of DL can be prioritized in stages where marginal gains in accuracy offer the greatest clinical and operational return (e.g., triage, early detection, trial enrichment), while more interpretable ML or rule-based tools can be utilized in contexts where explicit rationale and guideline alignment are paramount. At the same time, rigorous data curation, harmonized preprocessing, and robust external validation shift part of the “trust burden” from the internal mechanics of DL toward the quality of its development and evaluation pipeline. Interpretability should thus be treated as a continuum—global, local, and systems-level—rather than a binary property, and optimized alongside accuracy. In this governance-oriented perspective, the superior performance of DL serves as a fundamental starting point for the design of safe, accountable, and clinically acceptable deployment strategies. It does not represent the sole criterion for adoption.

A comprehensive review of the studies revealed substantial heterogeneity, attributable to variations in AI methodologies, sample sizes, and imaging modalities (22).

The results of these subgroup meta-analyses of various ML approaches (such as SVM, LR, XGBoost, etc.) demonstrate the variability in diagnostic performance across the different ML methods, which could introduce heterogeneity in our meta-analysis (23). With regard to the issue of sample size, it is evident that none of the studies under review performed sample size calculations. This is a notable omission in the reports of the AI models (24). With respect to the imaging modalities employed, 2 studies utilized both PET and structural MRI to construct the AI model, while the remaining 7 relied exclusively on PET imaging. The observed heterogeneity is due to variations across the imaging modalities. This heterogeneity arises from the unique signal-to-noise characteristics and preprocessing requirements inherent to each modality (25). Systematic evaluations indicate that the performance of a particular AI algorithm can vary by over 15% when applied to different modalities, reflecting modality-specific challenges in feature discernment (26).

Moreover, while meta-regression attributed a proportion of the variance to these methodological factors, it is imperative to acknowledge that heterogeneity also inherently reflects the profound neurobiological diversity within AD. The wide prediction regions observed in the HSROC models are likely to capture meaningful biological signals driven by atypical presentations, mixed pathologies (such as concomitant cerebrovascular disease or limbic-predominant age-related TDP-43 encephalopathy), and underlying genetic variants (e.g., APOE status). It is recommended that future radiomics studies adopt a new approach, moving away from the current practice of treating neurobiological heterogeneity solely as a confounding variable. Instead, it is advised that radiomics studies actively integrate neurobiological heterogeneity as a key stratifying factor.

In order to mitigate the effects of heterogeneity caused by method and modality, it is essential to standardize and preprocess data rigorously across studies. Standardized data reduces confounding factors and enhances the ability to learn meaningful patterns and features from neuroimaging data using machine and deep learning (27). Earlier research indicated that preprocessing is vital for the reliability and validity of neuroimaging studies, particularly in the context of AD (28). The majority of studies included in this analysis have addressed this particular step. However, potential disadvantages such as time consumption and inaccuracy necessitate the refinement of these systems as AI technology evolves.

Beyond the characteristics of the data itself and the processes applied to its preparation, the training of the model represents another critical factor in determining the performance of AI and serves as a further source of heterogeneity among the studies conducted. A further significant variable is the process of model training. The ultimate performance of a model is inextricably linked to its training methods. The decisions made during hyperparameter tuning, the total number of epochs, and the specific optimization routines employed all influence the bias-variance balance. The incorporation of early stopping, cross-validation, and adaptive scheduling remains the optimal approach for navigating the trade-off between underfitting and overfitting, thereby ensuring the development of robust, generalizable models (29–31).

External validation critically influences both the risk of bias and the generalizability of AI diagnostic studies. Studies across diverse clinical domains consistently demonstrate that algorithms evaluated only on internal (development) data often exhibit optimistic performance estimates and harbor undetected biases. In contrast, rigorous external validation—testing models on wholly independent cohorts—reveals performance degradation in 40 to 70% of cases. This process uncovers overfitting, spectrum bias, and population shifts that undermine generalizability and clinical trust (32, 33). Only two of the included studies applied external validation. Consequently, the lack of external validation in AI diagnostic accuracy studies may lead to a high risk of bias (34). When algorithms are not externally validated, overfitting to the idiosyncrasies of the development dataset can yield inflated metrics. This reflects spectrum bias and results in over-optimistic performance (33, 35). Also, performance variability is often revealed by external validation across different sites or time periods (32). However, traditional validation on independent cohorts remains fundamentally insufficient for clinical AI. It is imperative that future research incorporates a multidimensional evaluation framework, encompassing the following: (1) cross-demographic validation to ensure equity across underrepresented groups (36); (2) cross-site validation to confirm robustness against variations in scanners and protocols (37, 38); (3) validation within unselected, real-world clinical populations rather than highly curated research volunteers (39); and (4) validation across the continuous disease spectrum, including atypical presentations. All included studies are based on retrospective hospital data, which often yields overly optimistic performance estimates but limits real-world applicability (40, 41). In contrast, prospective studies—by evaluating algorithms on data collected after model development—provide more robust evidence that better aligns anticipated performance with actual clinical impact (42).

Amyloid-PET and tau-PET are imaging modalities that have been demonstrated to visualize hallmark AD pathologies in living subjects. These modalities offer both molecular specificity and quantifiable binding metrics (43). FDG-PET has been shown to be a reliable measure of cerebral glucose metabolism, serving as a surrogate marker for neurodegeneration. In many cases, its sensitivity has been found to exceed that of structural MRI (44).

A comparison of amyloid-PET and tau-PET visual reads reveals that both methods demonstrate similar sensitivity and specificity for the detection of AD in patients with cognitive impairment (45). In the context of multi-cohort studies, no statistically significant disparities in diagnostic precision were identified among the A/T/N, A/T, A, and T biomarkers when utilized for the identification of AD. This observation suggests that standalone PET measures may, under certain circumstances, approximate the A/T/N composite for specific clinical outcomes (46). Therefore, the categorization was conducted as such: the group consisting of *β*-amyloid biomarker imaging and tau biomarker imaging was designated as one category, and the group consisting of ^18^F-FDG imaging was designated as another category. In the previous studies, the AUC values of amyloid- PET and tau-PET were found to be highly accurate, with values ranging from 0.89 to 0.96 (47) and 0.92 to 0.94 (48), respectively.

While conventional ^18^F-FDG PET produces an AUC of 0.94 for AD vs. HC, specialized ^18^F-FDG PET DAT-score classifiers report AUCs around 0.78–0.81 for AD trajectory prediction (49). In comparison to the findings of this study, the overall AUC for proteinopathy is 0.96, while the overall AUC for ^18^F-FDG is 0.94 for the diagnosis of AD from NC. The overall AUC for proteinopathy is 0.96, while the overall AUC for ^18^F-FDG is 0.84 when diagnosing AD from MCI. A subset of these datasets exhibits substantial enhancement compared to previous datasets, while other datasets demonstrate relative consistency with prior datasets.

This review prioritized English-language publications, which may have excluded relevant non-English evidence. We did not contact study authors for missing data. Most included studies diagnosed AD using clinical criteria rather than pathological confirmation. Cohort compositions varied, including differing distributions of AD subtypes, which limited comparability. The evidence base was too sparse to explore sources of between-study heterogeneity. Limited PET data increases the risk of model overfitting, and cross-dataset generalizability remains uncertain. All cross-modality comparisons are indirect (across studies) rather than head-to-head within the same participants; residual confounding due to thresholds, reference standards, and case-mix heterogeneity may persist despite bivariate modeling. Future work should validate AI performance in real-world settings and in more homogeneous patient groups.

A key limitation of the current literature and this meta-analysis is the severe lack of population diversity, which is an unmeasured moderator that fundamentally limits the generalizability of our findings. It is important to note that seven of the nine included studies utilized data from the ADNI database, a cohort known to be predominantly non-Hispanic White and highly educated (50), thus not accurately reflecting the global AD demographic landscape. AI diagnostic models trained on homogeneous populations often experience significant performance degradation when applied to underrepresented or diverse groups. As a result, the clinical usefulness of current AI-radiomics models remains limited. It is essential that future validation studies not only report comprehensive demographic characteristics but also actively validate algorithms in populations that are truly representative of the intended clinical use settings.

Finally, some primary subgroup analyses included multiple data records (e.g., training, validation and test sets) from the same cohorts within individual studies, introducing within-study dependence. To address this, an independent sensitivity analysis was performed alongside a series of descriptive sensitivity checks for heavily restricted subgroups (N < 4). While it must be acknowledged that these descriptive checks lack the power for formal confirmation, they do generally align with and support the robustness of our primary pooled estimates. The employment of AI algorithms in the detection of AD through PET molecular imaging offers a promising outlook for advancements in the field of nuclear medicine. This includes a number of advanced applications, such as end-to-end deep learning classification, machine learning integration with early-phase protocols, and automated quantification software (51–53). Despite data governance and regulatory constraints, AI-assisted PET shows promise for diagnostic support. Implementation should prioritize external validation, decision-impact evaluation, and cost-effectiveness alongside accuracy.

## Data Availability

The original contributions presented in the study are included in the article/Supplementary material, further inquiries can be directed to the corresponding author.
