# Supplementary material for: Stage-stratified benefits of AI-radiomics PET in early Alzheimer’s disease: a systematic review and meta-analysis
Source: Front Neurol. 2026 May 15;17:1771993. doi: 10.3389/fneur.2026.1771993 (PMC13218884; doi:10.3389/fneur.2026.1771993)
Supplement: Supplementary file 1 [file Supplementary_file_1.docx]

Supplementary Material

| Author. Year | Participant demographics | | | | | |  | Data characteristics | | | | | Algorithm details | |
| --- | --- | --- | --- | --- | --- | --- | --- | --- | --- | --- | --- | --- | --- | --- |
|  | Target condition | Structural brain lesions | Other degenerative diseases excluded | Prior exposure to neuroleptics/ drug use excluded | Neurological / psychiatric illness excluded? | Reference standard | Pathological diagnosis | Imaging agent | Tracer type | Structural MRI? | Exclusion of poor-quality imaging | Open access data | AI vs visual interpretation? |  |
| Jiang et al., 2024 | AD vs. MCI | NR | NR | NR | NR | Expert consensus; follow-up | NR | ^18^F-FDG | Glucose metabolism | NR | NR | Yes | No |  |
| Mu et al., 2024 | AD vs. MCI | NR | NR | NR | NR | Expert consensus; three years follow-up | NR | ^18^F-FDG | Glucose metabolism | No | NR | Yes | NR |  |
| Chen et al., 2024 | AD vs. MCI;  AD vs. HC | Yes | NR | NR | Yes | Expert consensus | NR | ^18^F-FDG | Glucose metabolism | Yes | NR | No | NR |  |
| Peng et al., 2023 | AD vs. MCI | NR | NR | NR | NR | Expert consensus; follow-up | No | ^18^F-FDG | Glucose metabolism | Yes | Yes | Yes | NR |  |
| Alongi et al., 2022 | AD vs. HC | Yes | NR | NR | NR | Expert consensus; two years follow-up | No | ^18^F-FDG | Glucose metabolism | NR | NR | No | NR |  |
| Zhao et al., 2022 | AD vs. MCI;  AD vs. HC | NR | NR | NR | NR | Expert consensus | No | ^18^F-AV1451 | Tau PET | Yes | Yes | Yes | NR |  |
| Ding et al., 2021 | AD vs. HC | NR | NR | NR | NR | Expert consensus; four years follow-up | No | ^18^F-AV45 | Amyloid PET | Yes | NR | Yes | NR |  |
| Zhou et al., 2021 | AD vs. MCI | NR | NR | NR | NR | Expert consensus; three years follow-up | No | ^18^F-FDG | Glucose metabolism | No | NR | Yes | NR |  |
| Dai et al., 2021 | AD vs. HC | NR | NR | NR | NR | Expert consensus | No | ^18^F-FDG | Glucose metabolism | Yes | NR | Yes | NR |  |

Amyloid PET: ^11^C-PiB, ^18^F-florbetapir or CSF Aβ42 levels; Tau PET: ^18^F-AV-1451 or CSF p-tau181; Glucose metabolism: ^18^F-FDG; AD: Alzheimer Disease; HC: Healthy Control; MCI: Mild Cognitive Impairment; ^18^F-AV1451: ^18^F-flortaucipir; ^18^F-AV45: ^18^F-florbetapir; ^18^F-FDG: ^18^F-fluorodeoxyglucose; NR: not reported; AI: artificial intelligence; MRI: magnetic resonance imaging

**Supplementary Table 1.** Other characteristics of all included studies (n = 9)

| Category | | Number of studies | Number of tables |
| --- | --- | --- | --- |
| PD diagnosis classification | AD from HC | 5 | 10 |
|  | AD from MCI | 7 | 11 |
| PET imaging agent | proteinopathy imaging |  |  |
|  | 18F‑AV1451 (tau‑PET) | 1 | 4 |
|  | 18F‑AV45 (amyloid‑PET) | 1 | 3 |
|  | neurodegeneration imaging |  |  |
|  | 18F-FDG | 7 | 18 |
| AI algorithm | deep learning | 4 | 10 |
|  | machine learning |  |  |
|  | GBDT | 2 | 4 |
|  | Logistic Regression | 2 | 6 |
|  | mRMR | 2 | 6 |
|  | LASSO | 1 | 4 |
|  | Support Vector Machine | 1 | 3 |
|  | Domain Adaptation | 1 | 4 |

**Supplementary Table 2.** Detailed enumeration for various categories across different PET tracers

| Imaging agent | Category | Number of studies | Number of tables |
| --- | --- | --- | --- |
| Proteinopathy PET imaging | Proteinopathy PET imaging | 2 | 5 |
|  | tau‑PET | 1 | 2 |
|  | amyloid‑PET | 1 | 3 |
|  | Algorithm |  |  |
|  | DL | 1 | 2 |
|  | ML | 1 | 3 |
|  | Sample size |  |  |
|  | ≥100 | 2 | 4 |
|  | <100 | 0 | 1 |
|  | Structural MRI |  |  |
|  | Yes | 0 | 0 |
|  | No | 0 | 0 |
| Neurodegeneration imaging | ^18^F-FDG | 3 | 5 |
|  | Algorithm |  |  |
|  | DL | 2 | 3 |
|  | ML | 1 | 2 |
|  | Sample size |  |  |
|  | ≥100 | 2 | 3 |
|  | <100 | 1 | 2 |
|  | Structural MRI |  |  |
|  | Yes | 1 | 2 |
|  | No | 0 | 0 |

**Supplementary Table 3.** Detailed enumeration of various categories across different PET tracers (AD vs. HC)

| Imaging agent | Category | Number of studies | Number of tables |
| --- | --- | --- | --- |
| Proteinopathy PET imaging | Proteinopathy PET imaging | 1 | 2 |
|  | tau‑PET | 1 | 2 |
|  | amyloid‑PET | 0 | 0 |
|  | Algorithm |  |  |
|  | DL | 1 | 2 |
|  | ML | 0 | 0 |
|  | Sample size |  |  |
|  | ≥100 | 1 | 1 |
|  | <100 | 0 | 1 |
|  | Structural MRI |  |  |
|  | Yes | 0 | 0 |
|  | No | 0 | 0 |
| Neurodegeneration imaging | ^18^F-FDG | 6 | 13 |
|  | Algorithm |  |  |
|  | DL | 2 | 3 |
|  | ML | 4 | 10 |
|  | Sample size |  |  |
|  | ≥100 | 3 | 5 |
|  | <100 | 3 | 8 |
|  | Structural MRI |  |  |
|  | Yes | 1 | 2 |
|  | No | 0 | 0 |

**Supplementary Table 4.** Detailed enumeration of various categories across different PET tracers (AD vs. MCI)

| Category | No. of tables | AUC (95% CI) | Sensitivity (%) | | Specificity (%) | |
| --- | --- | --- | --- | --- | --- | --- |
|  |  |  | SE (95% CI) | I^2^ (95% CI) | SP (95% CI) | I^2^ (95% CI) |
| LR | 4 | 0.88 (0.78-0.90) | 89 (79-94) | 0 | 70 (55-81) | 69 (0-81) |
| RF | 2 | 0.88 (0.83-0.88) | 86 (79-92) | 0 | 79 (73-84) | 0 |
| DA | 4 | 0.78 (0.69-0.86) | 76 (65-84) | 0 | 68 (54-79) | 31 (0-44) |

**Supplementary Table 5.** Classification accuracy using different ML algorithms-assisted 18F-FDG PET imaging (AD vs. MCI)

| Category | No. of tables | AUC (95% CI) | Sensitivity (%) | | Specificity (%) | |
| --- | --- | --- | --- | --- | --- | --- |
|  |  |  | SE (95% CI) | I^2^ (95% CI) | SP (95% CI) | I^2^ (95% CI) |
| Structural MRI |  |  |  |  |  |  |
| Yes | 2 | 0.97 (0.95-0.98) | 89 (77-96) | 0 | 94 (98-98) | 0 |
| No | 3 | 0.94 (0.91-0.98) | 92 (98-95) | 0 | 92 (82-96) | 85 (0-89) |

**Supplementary Table 6.** Classification accuracy using sMRI assisted AI algorithm in 18F-FDG PET imaging AD vs. HC)

| Category | No. of tables | AUC (95% CI) | Sensitivity (%) | | Specificity (%) | |
| --- | --- | --- | --- | --- | --- | --- |
|  |  |  | SE (95% CI) | I^2^ (95% CI) | SP (95% CI) | I^2^ (95% CI) |
| Structural MRI |  |  |  |  |  |  |
| Yes | 2 | 0.89 (0.86-0.90) | 84 (72-92) | 0 | 81 (69-90) | 0 |
| No | 11 | 0.84 (0.80-0.88) | 82 (78-85) | 0 | 73 (66-80) | 71 (32-79) |

**Supplementary Table 7.** Classification accuracy using sMRI assisted AI algorithm in 18F-FDG PET imaging AD vs. MCI)

| Category | No. of tables | AUC (95% CI) | Sensitivity (%) | | Specificity (%) | |
| --- | --- | --- | --- | --- | --- | --- |
|  |  |  | SE (95% CI) | I^2^ (95% CI) | SP (95% CI) | I^2^ (95% CI) |
| ^18^F‑AV1451 (tau‑PET) | 2 | 0.98 (0.98-0.99) | 97 (92-99) | 0 | 99 (95-100) | 17 (0-20) |
| ^18^F‑AV45 (amyloid‑PET) | 3 | 0.89 (0.82-0.93) | 80 (69-87) | 91 (0-93) | 85 (79-89) | 78 (0-82) |

**Supplementary Table 8.** Classification accuracy using proteinopathy PET imaging (AD vs. HC)

| Author^[ref]^, year | Subject selection | | | | | | Index Test | | Reference standard | | Flow and Timing | |
| --- | --- | --- | --- | --- | --- | --- | --- | --- | --- | --- | --- | --- |
|  | Q1 | Q2 | Q3 | Q4 | Q5 | Risk | Q6 | Risk | Q7 | Risk | Q8 | Risk |
| Jiang et al.2024 | Yes | No | Yes | Yes | No | Low | Yes | Low | Yes | Low | Yes | Low |
| Mu et al.2024 | Yes | No | Yes | Yes | No | Low | No | High | Yes | Low | Yes | Low |
| Chen et al.2024 | Yes | No | Yes | Yes | No | Low | No | High | Yes | Low | Yes | Low |
| Peng et al.2023 | Yes | No | Yes | Yes | No | Low | No | High | Yes | Low | Yes | Low |
| Alongi et al.2022 | Yes | Yes | Yes | Yes | Yes | Low | No | High | Yes | Low | Yes | Low |
| Zhao et al.2022 | Yes | No | Yes | Yes | No | Low | No | High | Yes | Low | Yes | Low |
| Ding et al.2021 | Yes | No | Yes | Yes | No | Low | Yes | Low | Yes | Low | Yes | Low |
| Zhou et al.2021 | Yes | No | Yes | Yes | No | Low | No | High | Yes | Low | Yes | Low |
| Dai et al.2021 | Yes | No | Yes | Yes | No | Low | No | High | Yes | Low | Yes | Low |

QUADAS-AI (quality assessment of diagnostic accuracy studies-AI)

**Supplementary Table 9.** Tabular presentation of QUADAS-AI

| Study | Total | TP | FP | FN | TN | Model (feature set) | ML/DL | Comparison (positive/negative) | PET imaging agent |
| --- | --- | --- | --- | --- | --- | --- | --- | --- | --- |
| Jiang 2024 | 884 | 356 | 40 | 26 | 462 | IDLR | DL | AD vs HC | ^18^F-FDG |
| Jiang 2024 | 737 | 314 | 78 | 68 | 277 | IDLR | DL | AD vs MCI | ^18^F-FDG |
| Jiang 2024 | 304 | 72 | 9 | 9 | 214 | IDLR | DL | AD vs HC | ^18^F-FDG |
| Jiang 2024 | 122 | 69 | 4 | 12 | 37 | IDLR | DL | AD vs MCI | ^18^F-FDG |
| Mu 2024 | 222 | 44 | 56 | 4 | 118 | ROI_total (all 4 ROIs) | ML | AD vs MCI | ^18^F-FDG |
| Mu 2024 | 56 | 12 | 21 | 0 | 23 | ROI_total | ML | AD vs MCI | ^18^F-FDG |
| Chen 2024 | 74 | 32 | 1 | 4 | 37 | Multimodal | ML | AD vs HC | ^18^F-FDG |
| Chen 2024 | 75 | 30 | 6 | 6 | 33 | Multimodal | ML | AD vs MCI | ^18^F-FDG |
| Chen 2024 | 30 | 15 | 1 | 0 | 14 | Multimodal | ML | AD vs HC | ^18^F-FDG |
| Chen 2024 | 31 | 13 | 4 | 2 | 12 | Multimodal | ML | AD vs MCI | ^18^F-FDG |
| Peng 2023 | 238 | 62 | 36 | 9 | 131 | Integrated model | ML | AD vs MCI | ^18^F-FDG |
| Peng 2023 | 103 | 26 | 14 | 5 | 58 | Integrated model | ML | AD vs MCI | ^18^F-FDG |
| Alongi 2022 | 43 | 15 | 9 | 7 | 12 | Other | ML | AD vs MCI | ^18^F-FDG |
| Alongi 2022 | 43 | 17 | 4 | 5 | 17 | Other | ML | AD vs MCI | ^18^F-FDG |
| Alongi 2022 | 43 | 18 | 5 | 4 | 16 | Other | ML | AD vs MCI | ^18^F-FDG |
| Alongi 2022 | 43 | 17 | 9 | 5 | 12 | Other | ML | AD vs MCI | ^18^F-FDG |
| Zhao 2022 | 274 | 96 | 4 | 5 | 169 | DLR model | DL | AD vs MCI | tau‑PET |
| Zhao 2022 | 30 | 11 | 2 | 1 | 16 | DLR model | DL | AD vs MCI | tau‑PET |
| Zhao 2022 | 290 | 99 | 0 | 2 | 189 | DLR model | DL | AD vs HC | tau‑PET |
| Zhao 2022 | 32 | 12 | 0 | 0 | 20 | DLR model | DL | AD vs HC | tau‑PET |
| Ding 2021 | 625 | 250 | 40 | 41 | 294 | SVM | ML | AD vs HC | amyloid‑PET |
| Ding 2021 | 625 | 204 | 67 | 87 | 267 | SVM | ML | AD vs HC | amyloid‑PET |
| Ding 2021 | 625 | 236 | 47 | 55 | 287 | SVM | ML | AD vs HC | amyloid‑PET |
| Zhou 2021 | 34 | 14 | 1 | 2 | 17 | DLR+C model (proposed) | DL | AD vs MCI | ^18^F-FDG |
| Dai 2021 | 182 | 94 | 14 | 8 | 66 | DeU-Net | DL | AD vs HC | ^18^F-FDG |

**Supplementary Table 10.** Contingency tables (31 tables from 9 studies)

| Domain | Subject selection | Index text (AI) | Reference standard | Work-flow |
| --- | --- | --- | --- | --- |
| Concern | Signaling question:    Q1: Accurately characterize the source, size and quality of input data alongside clear patient eligibility criteria?    Q2: Was it derived from open-source datasets?    Q3: Present the rationale and breakdown of its training, validation and test sets?    Q4: Whether to perform image preprocessing?    Q5: Provide the scanner model information used to acquire imaging data? | Signaling question:    Q6: Was external verification performed? | Signaling question:    Q7: Was the reference standard likely to correctly classify the target condition? | Signaling question:    Q8: Was the time between the index test and the reference standard reasonable? |
| Concerns regarding “risk of bias” | Risk of bias is judged as “low”, “high”, or “unclear”. If all signaling questions for a domain are answered “yes” then risk of bias can be judged “low”.    If any signaling question is answered “no” this flags the potential for bias. Review authors then need to have in-depth discussions to judge the risk of bias.    The “unclear” category should be used only when insufficient data are reported to permit a judgment. | | | |

**Supplementary Table 11.** Description of quality assessment based on QUADAS-AI domains


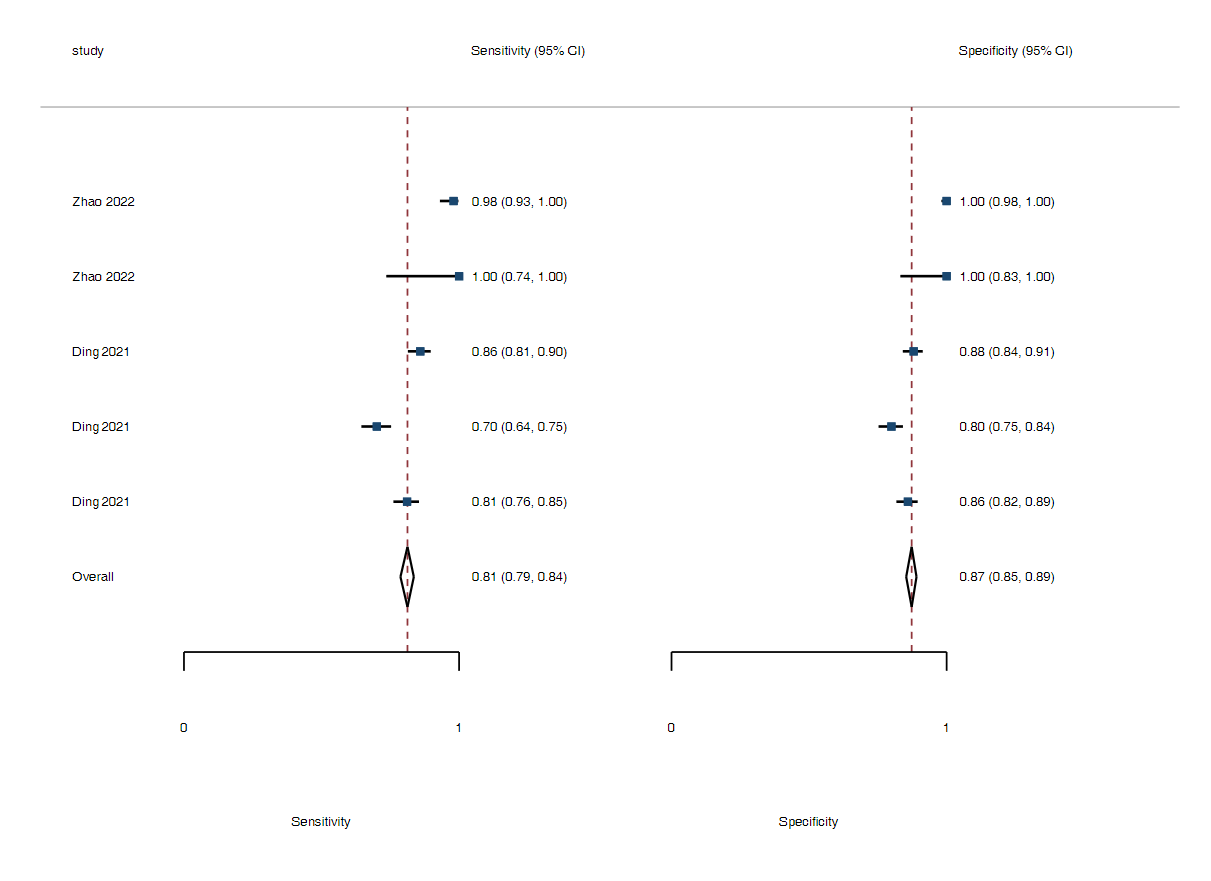


**Supplementary Figure 1.** Forest plot using proteinopathy PET imaging (AD vs. HC)


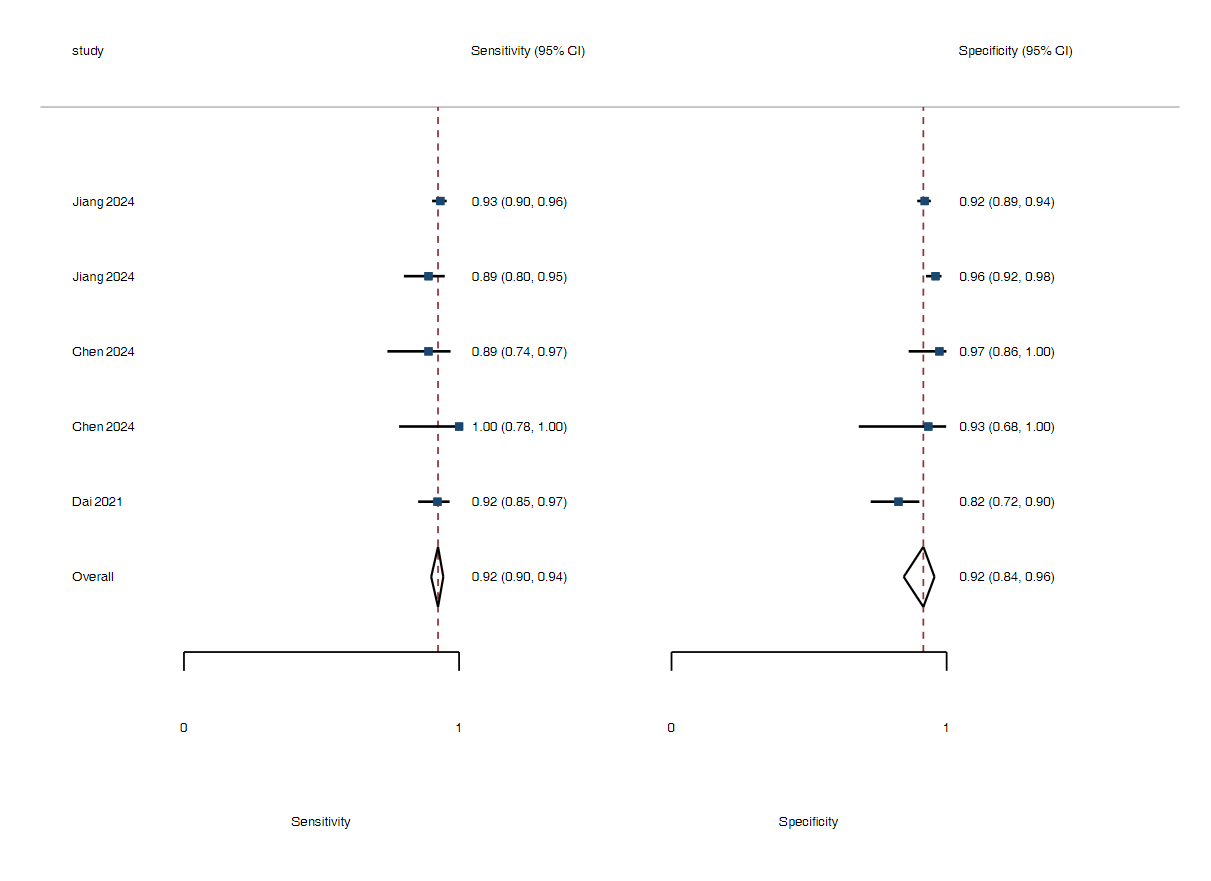


**Supplementary Figure 2.** Forest plot using ^18^F-FDG PET imaging (AD vs. HC)


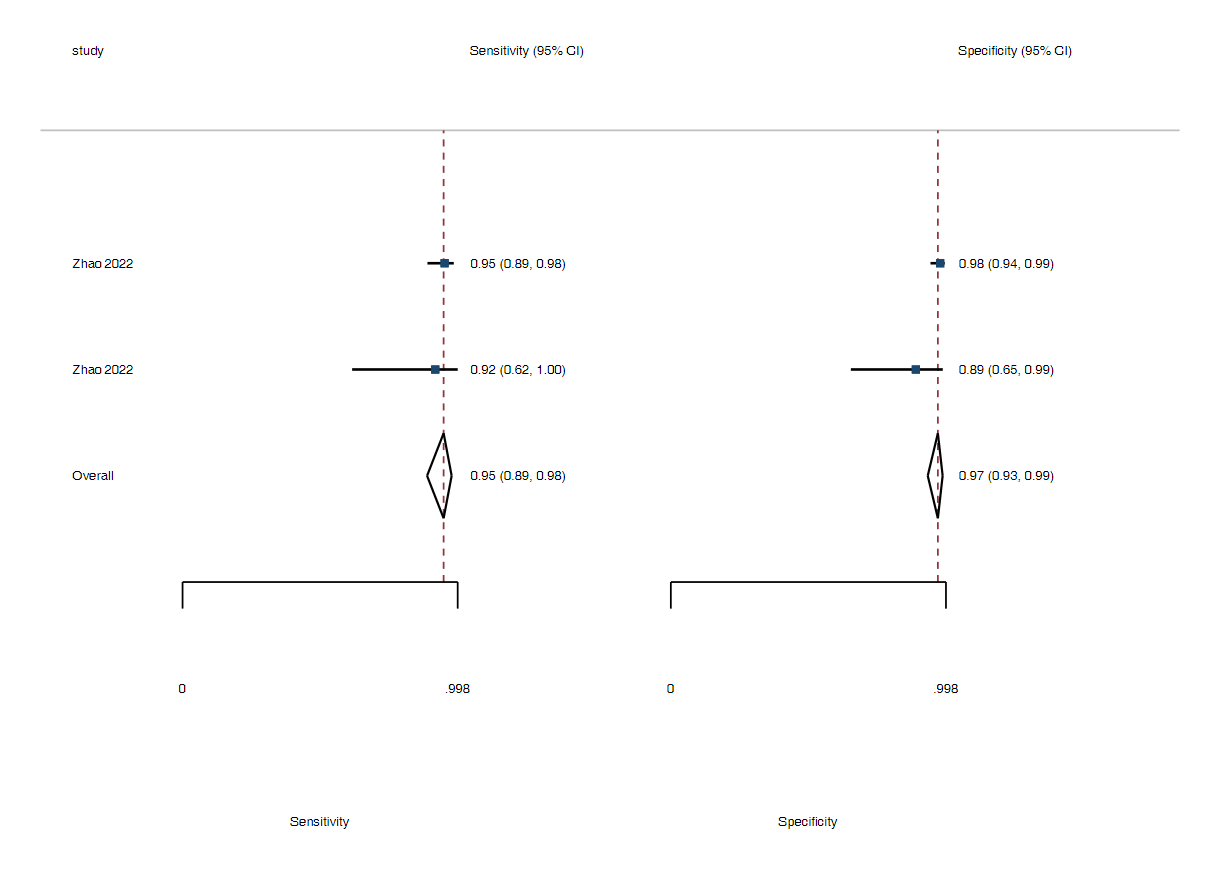


**Supplementary Figure 3.** Forest plot using proteinopathy PET imaging (AD vs. MCI)


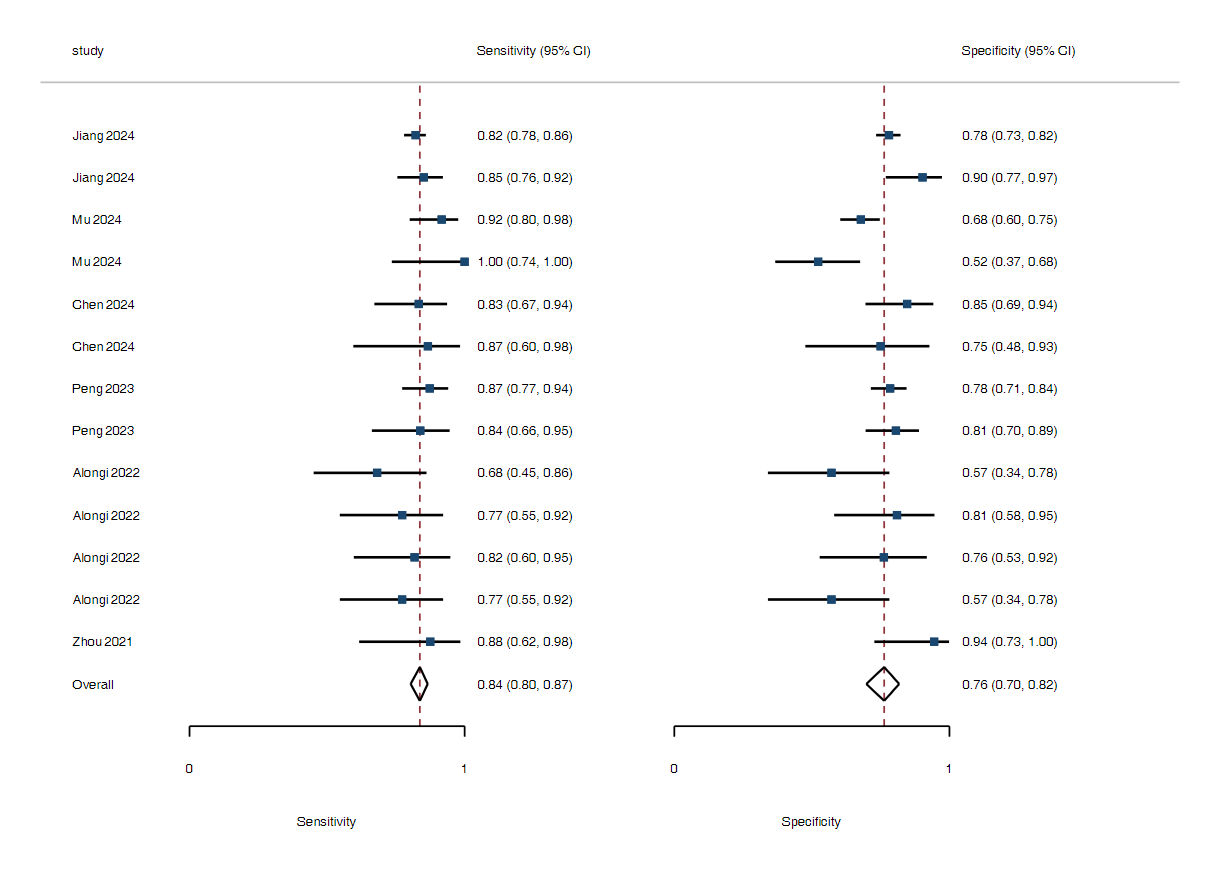


**Supplementary Figure 4.** Forest plot using ^18^F-FDG PET imaging (AD vs. MCI)


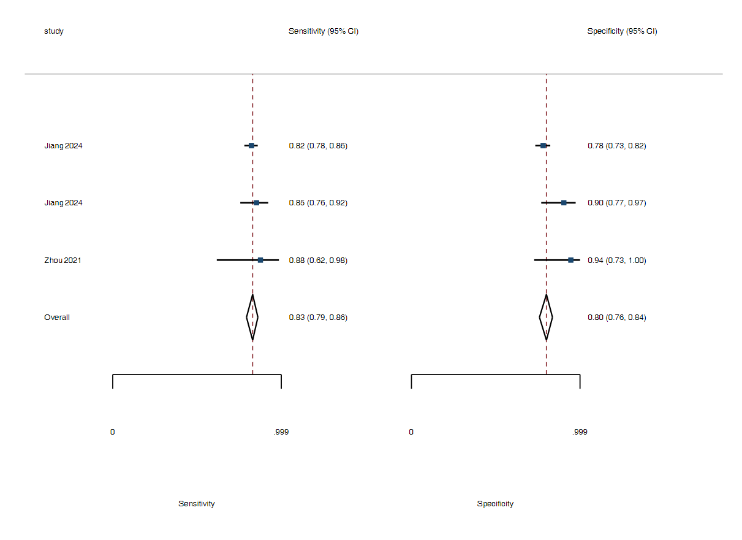


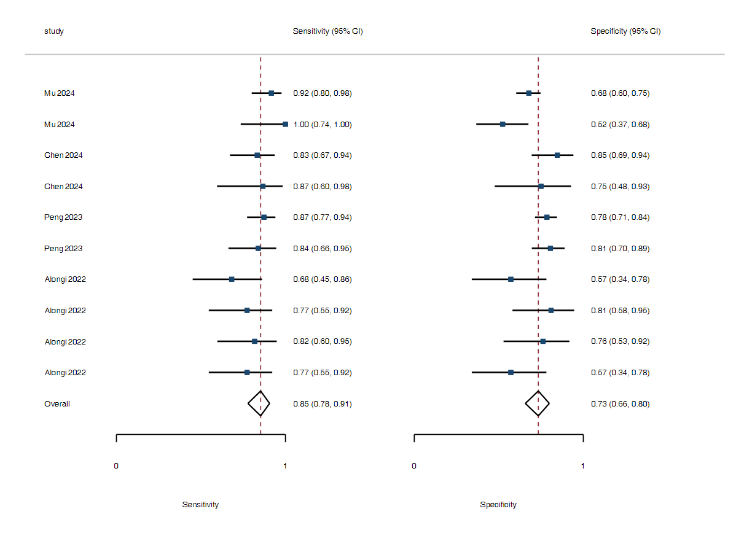


**Supplementary Figure 5.** Forest plot using DL- and ML-assisted 18F-FDG PET imaging (AD vs. MCI). **Figure 5.1** ML (6 studies with 63 contingency tables). **Figure 5.2** DL (4 studies with 53 contingency tables).


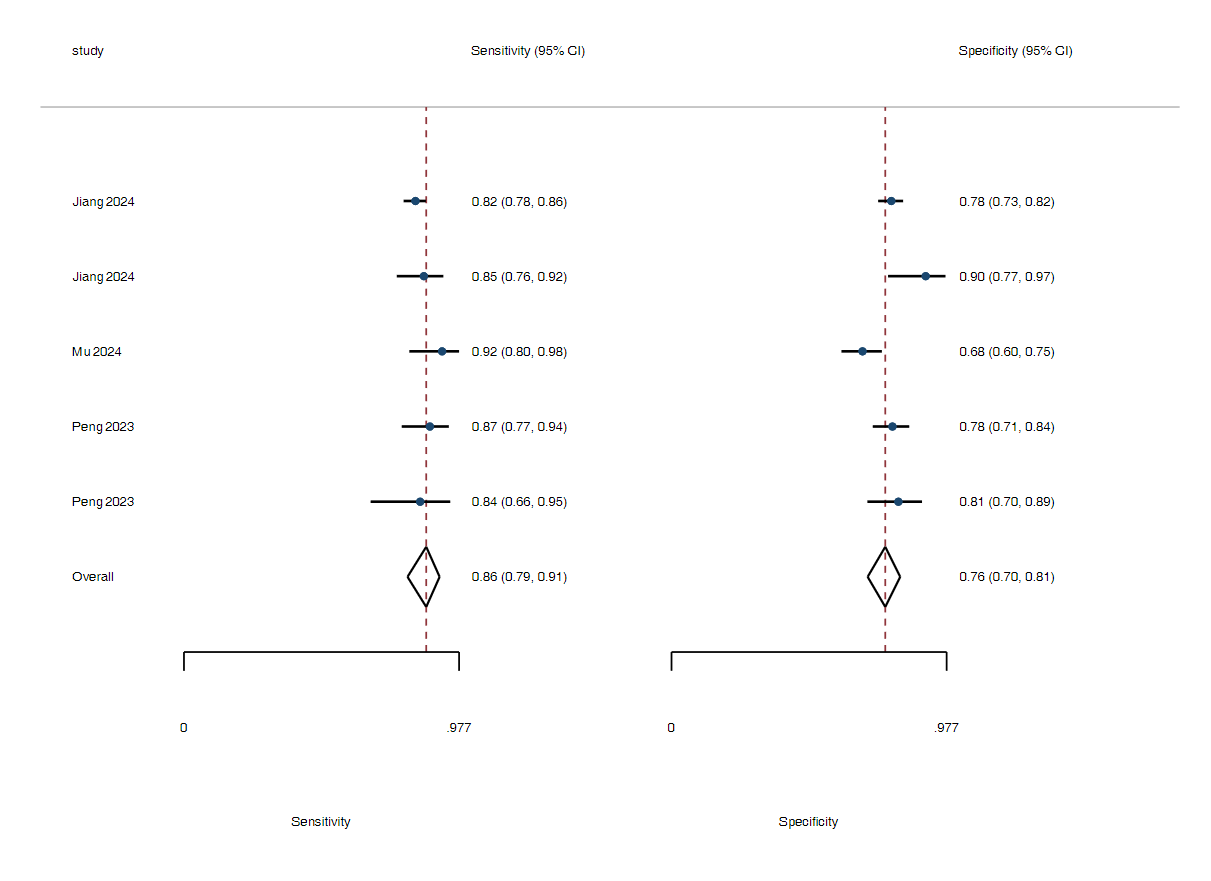


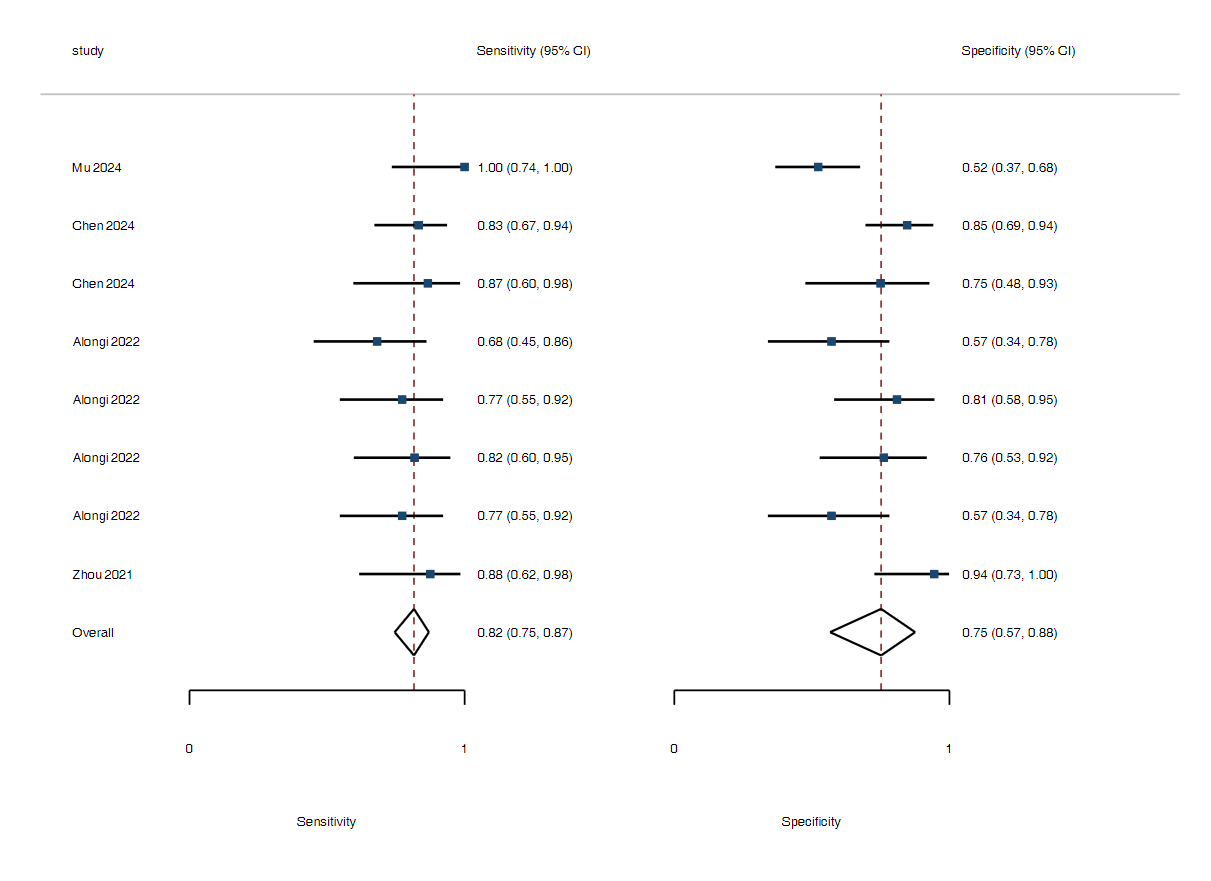


Supplementary Figure 6. Forest plot using ^18^F-FDG PET imaging based on sample size (AD vs. MCI) Figure 6.1 sample size ≥ 100 Figure 6.2 sample size < 100


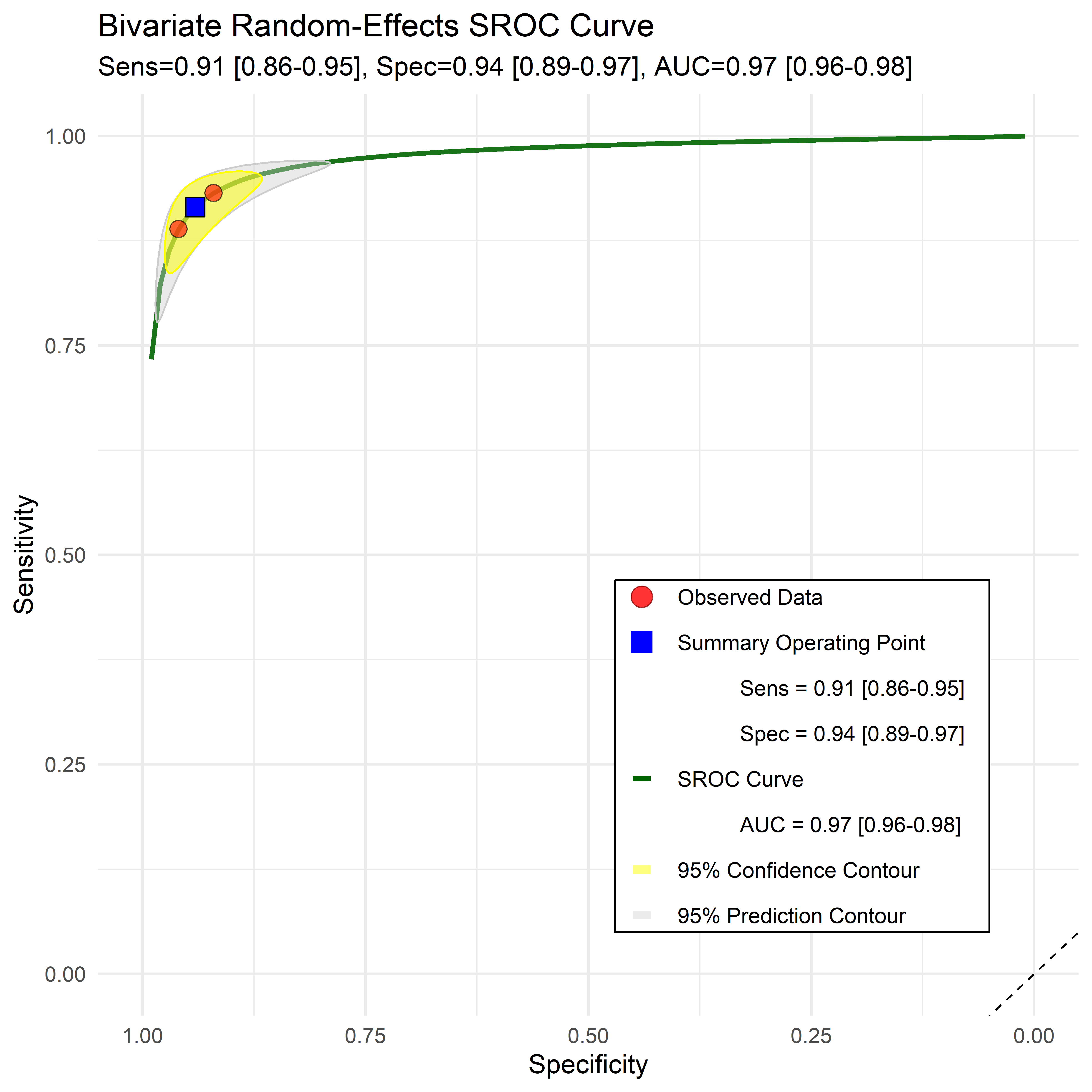


**Supplementary Figure 7.** HSROC curves (bivariate model) in optimistic scenario using ^18^F-FDG PET imaging (AD vs. HC)


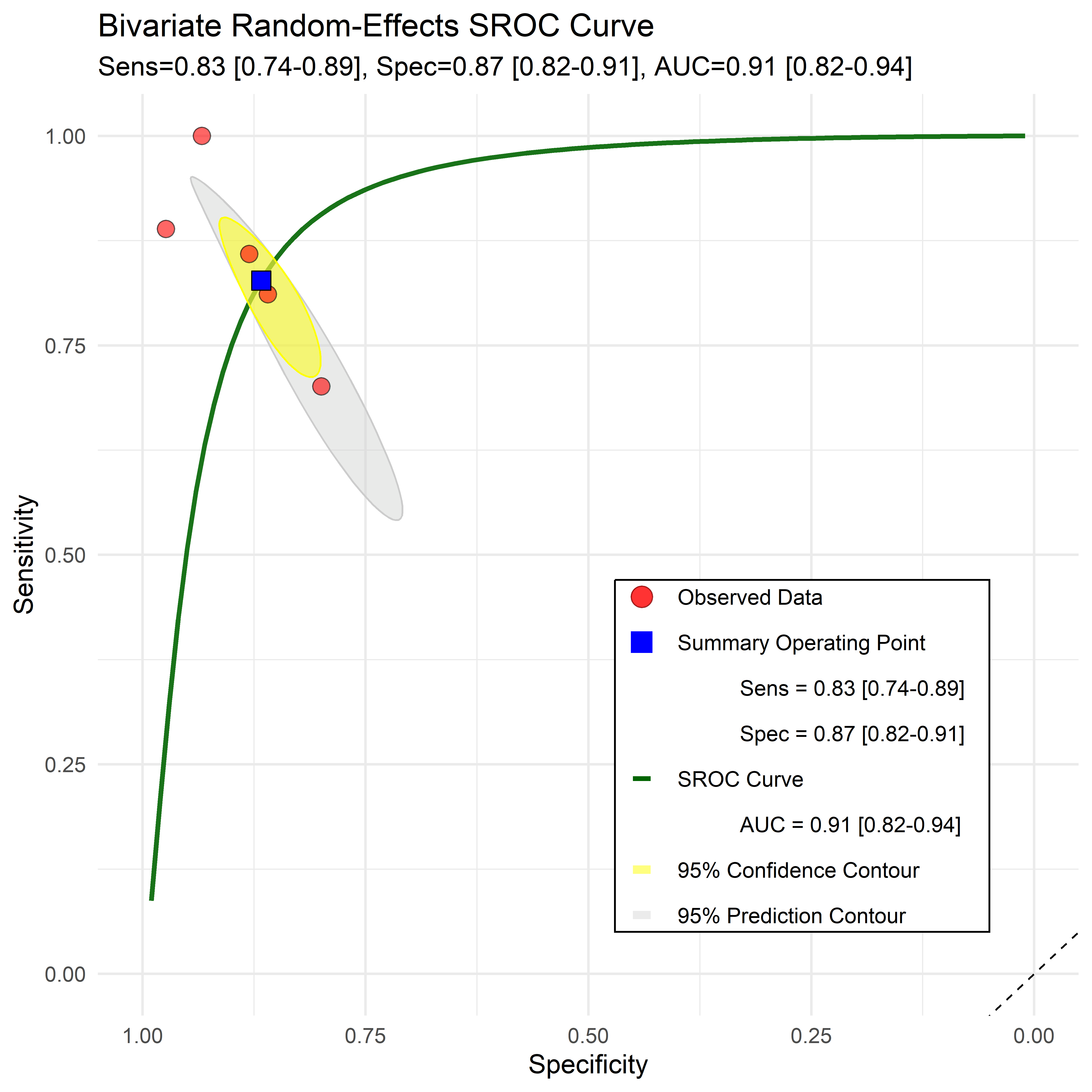

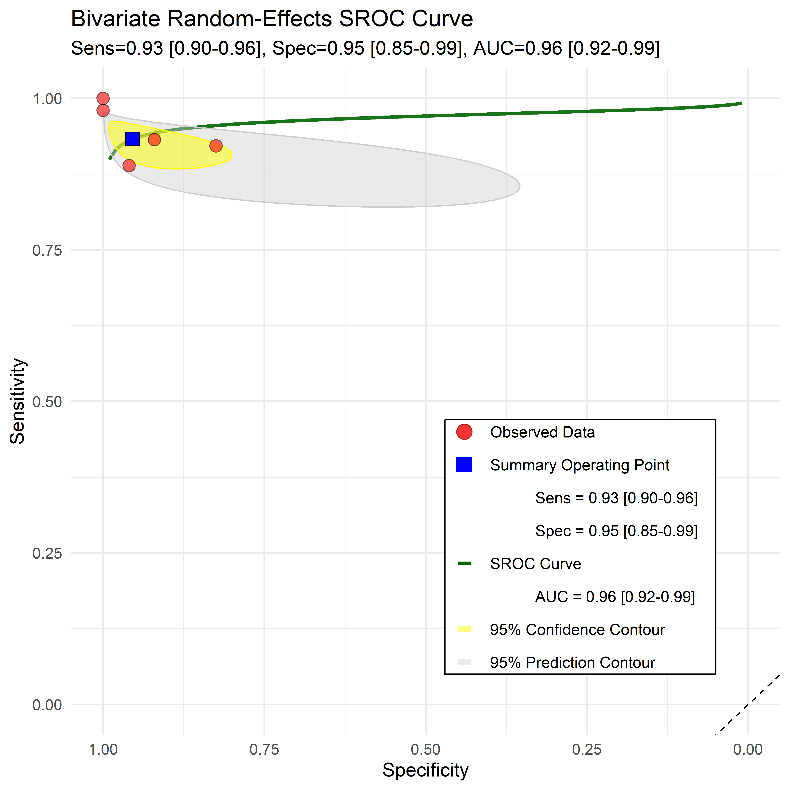


**Supplementary Figure 8.** HSROC curves (bivariate model) using DL- and ML-assisted ^18^F-FDG PET imaging (AD vs. HC) **Figure 8.1** DL **Figure 8.2** ML


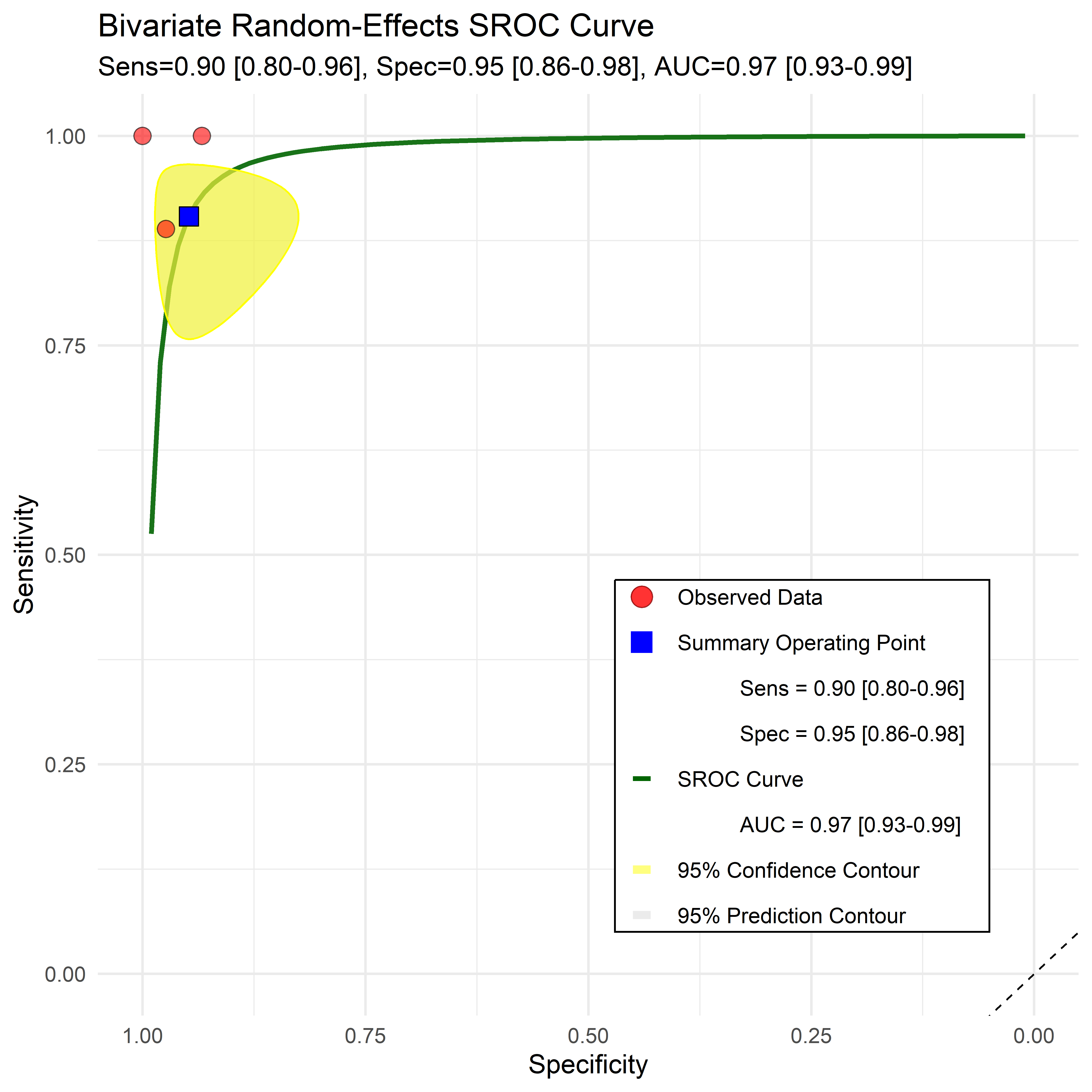

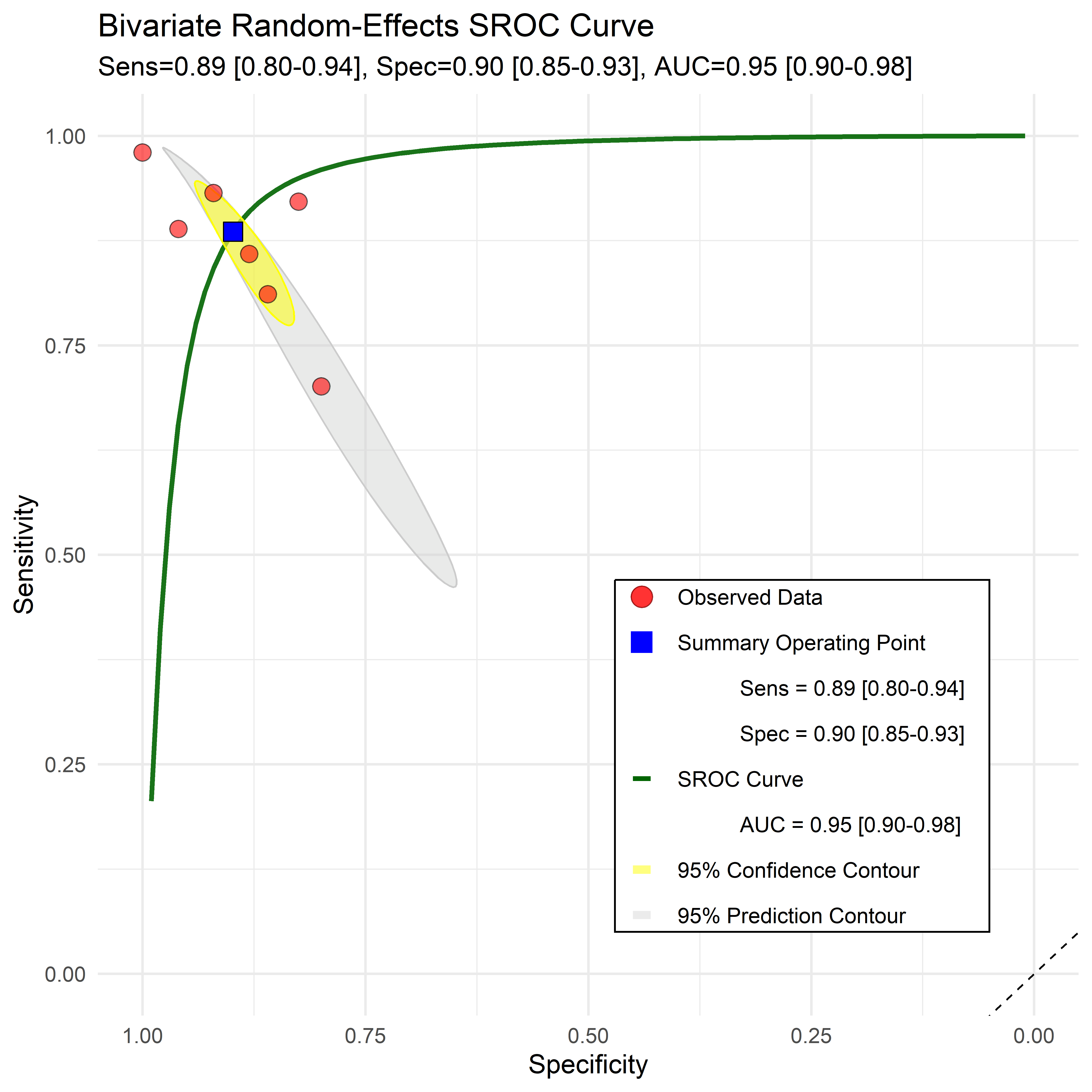


9 b

< 100

9 a

≥ 100

**Supplementary Figure 9.** HSROC curves (bivariate model) using ^18^F-FDG PET imaging based on sample size (AD vs. HC) **Figure 9.1** Sample size ≥ 100 **Figure 9.2** Sample size < 100


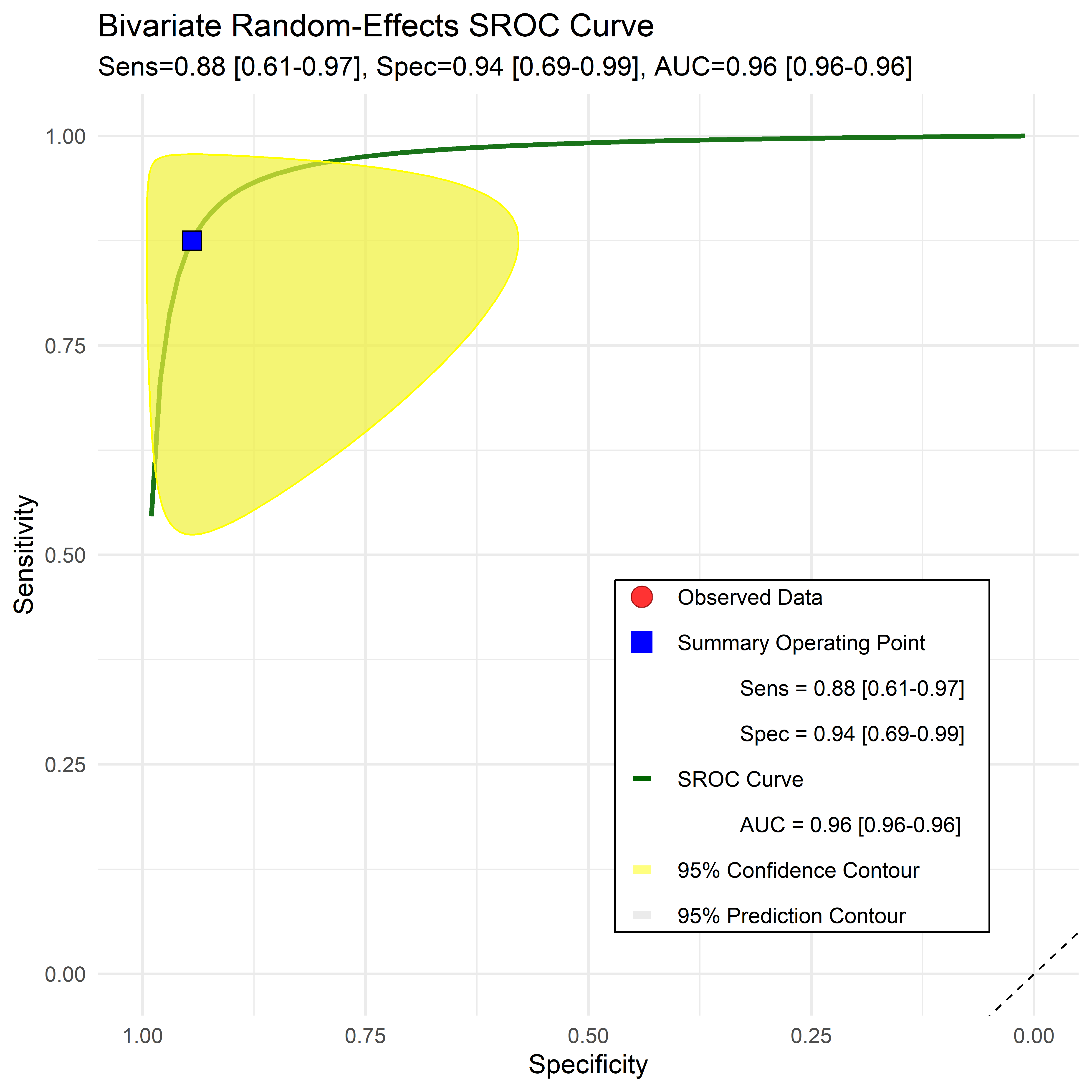


**Supplementary Figure 10.** HSROC curves (bivariate model) in optimistic scenario using ^18^F-FDG PET imaging (AD vs. MCI)


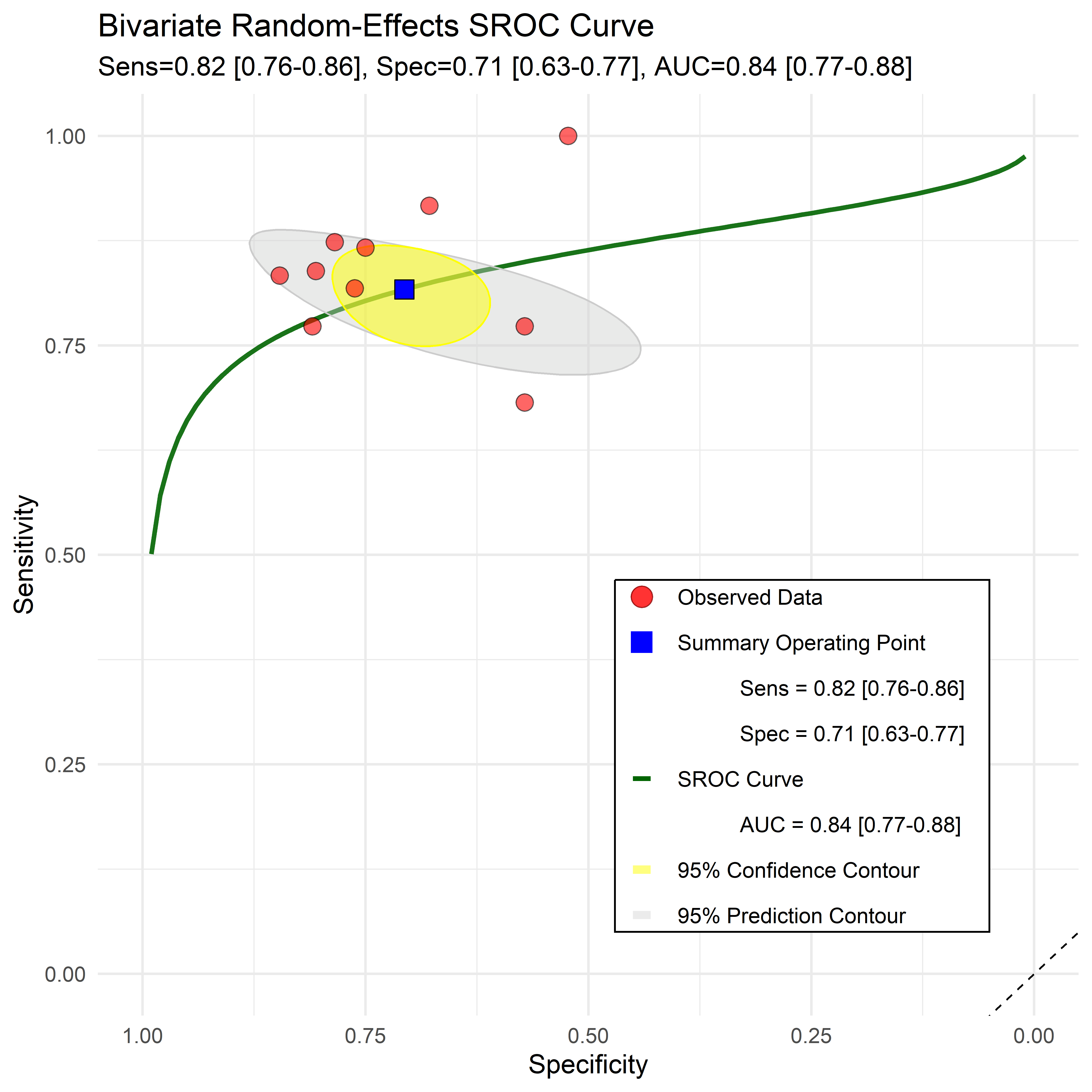

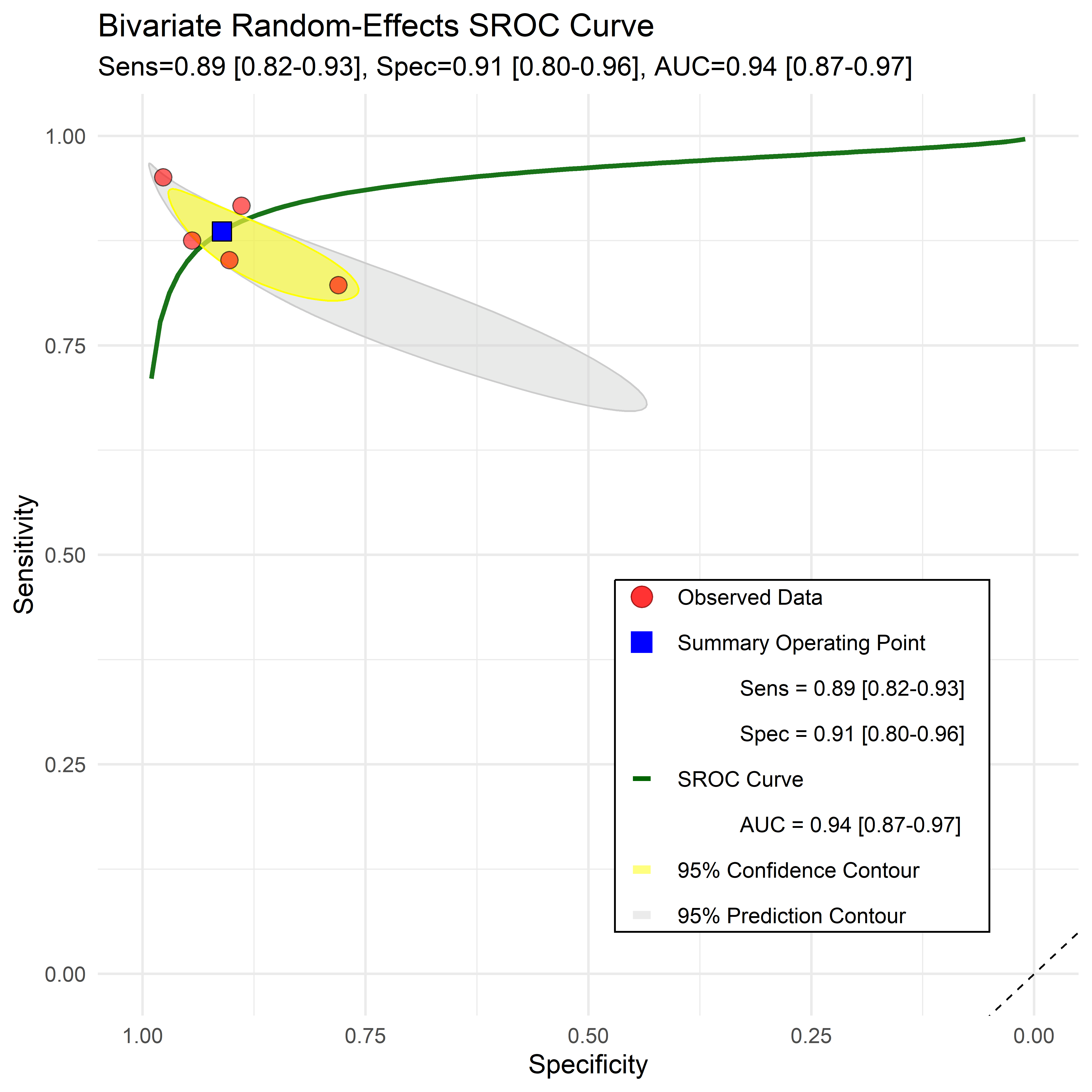
**Supplementary Figure 11.** HSROC curves (bivariate model) using DL- and ML-assisted ^18^F-FDG PET imaging (AD vs. MCI) **Figure 11.1** DL **Figure 11.2** ML


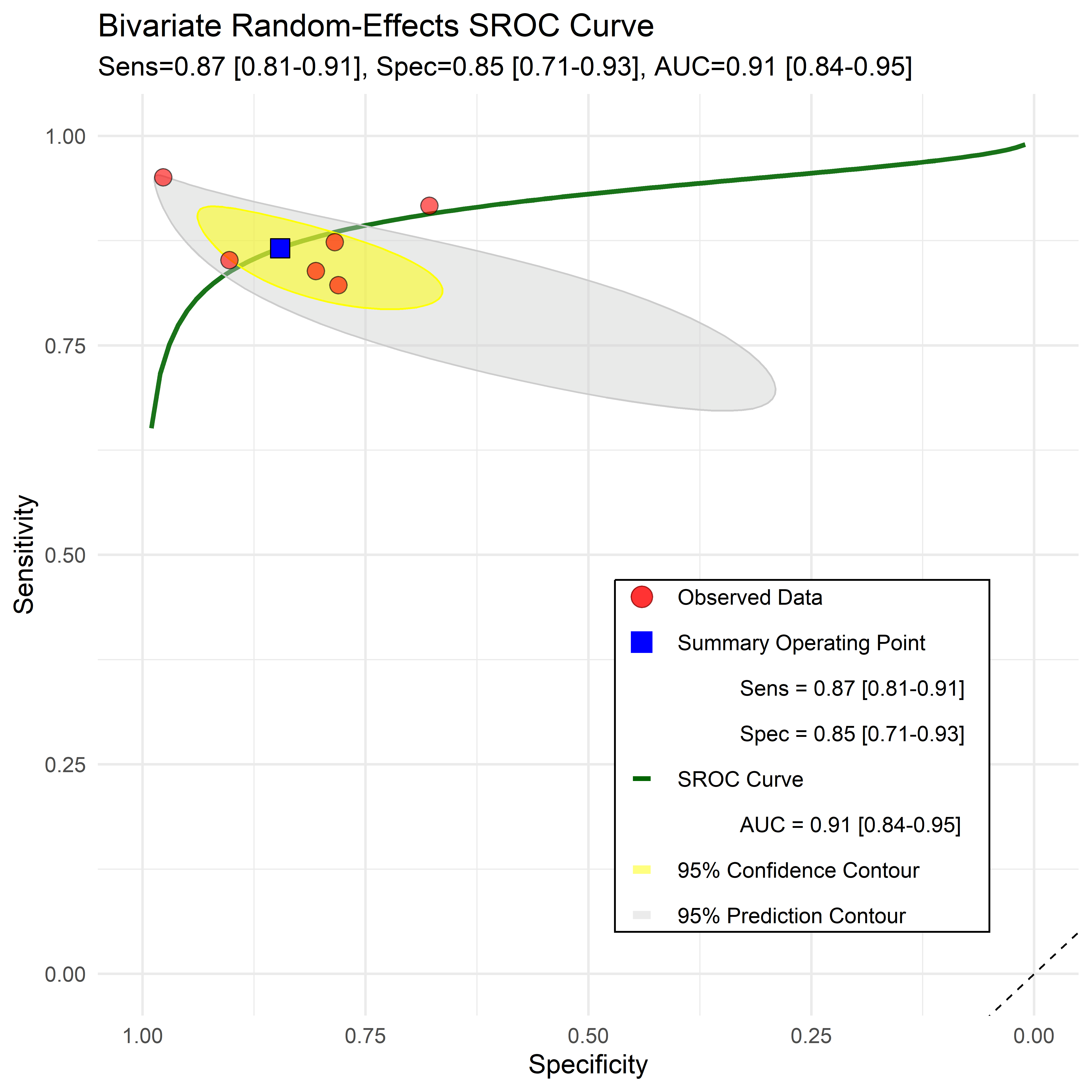

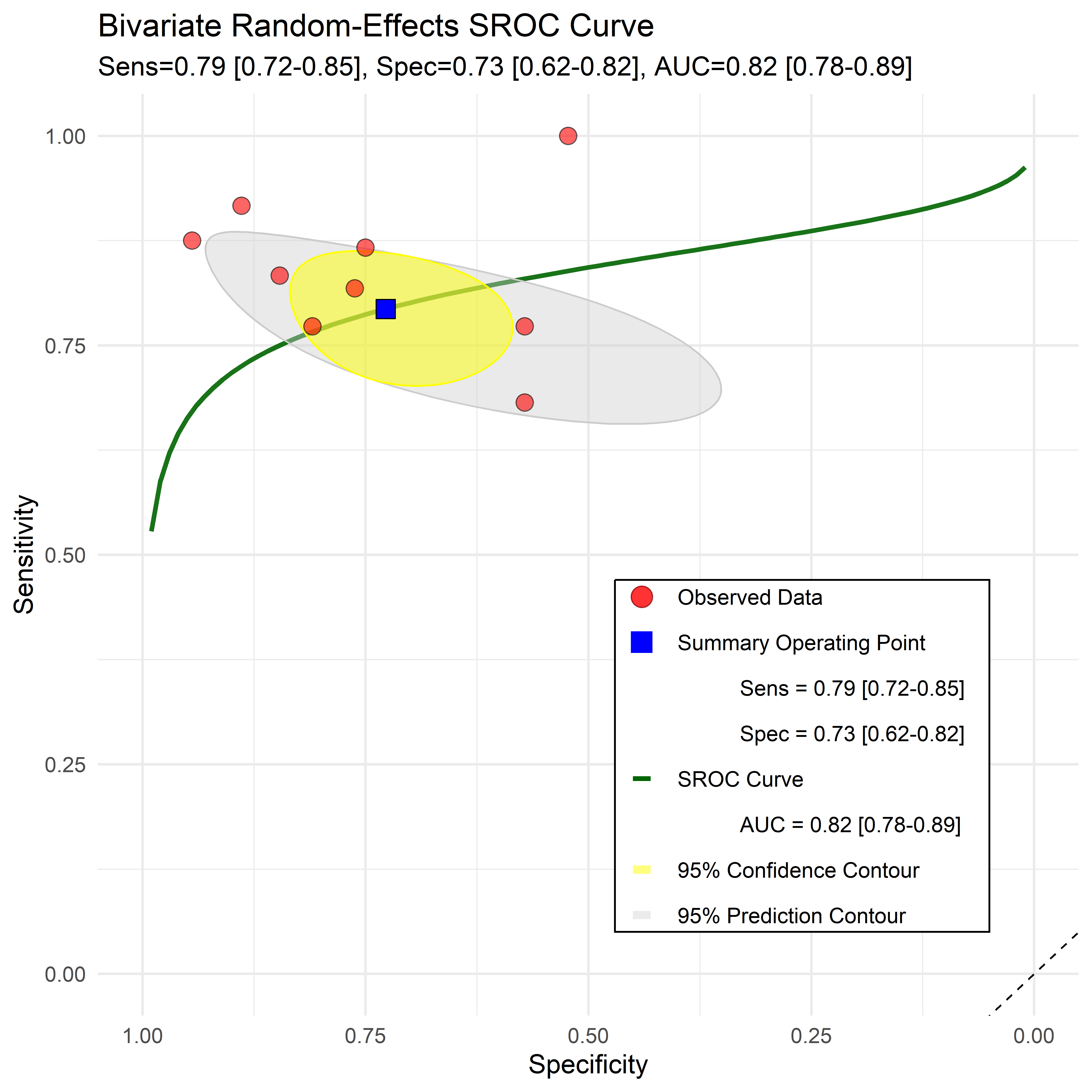
**Supplementary Figure 12.** HSROC curves (bivariate model) using ^18^F-FDG PET imaging based on sample size (AD vs. MCI)


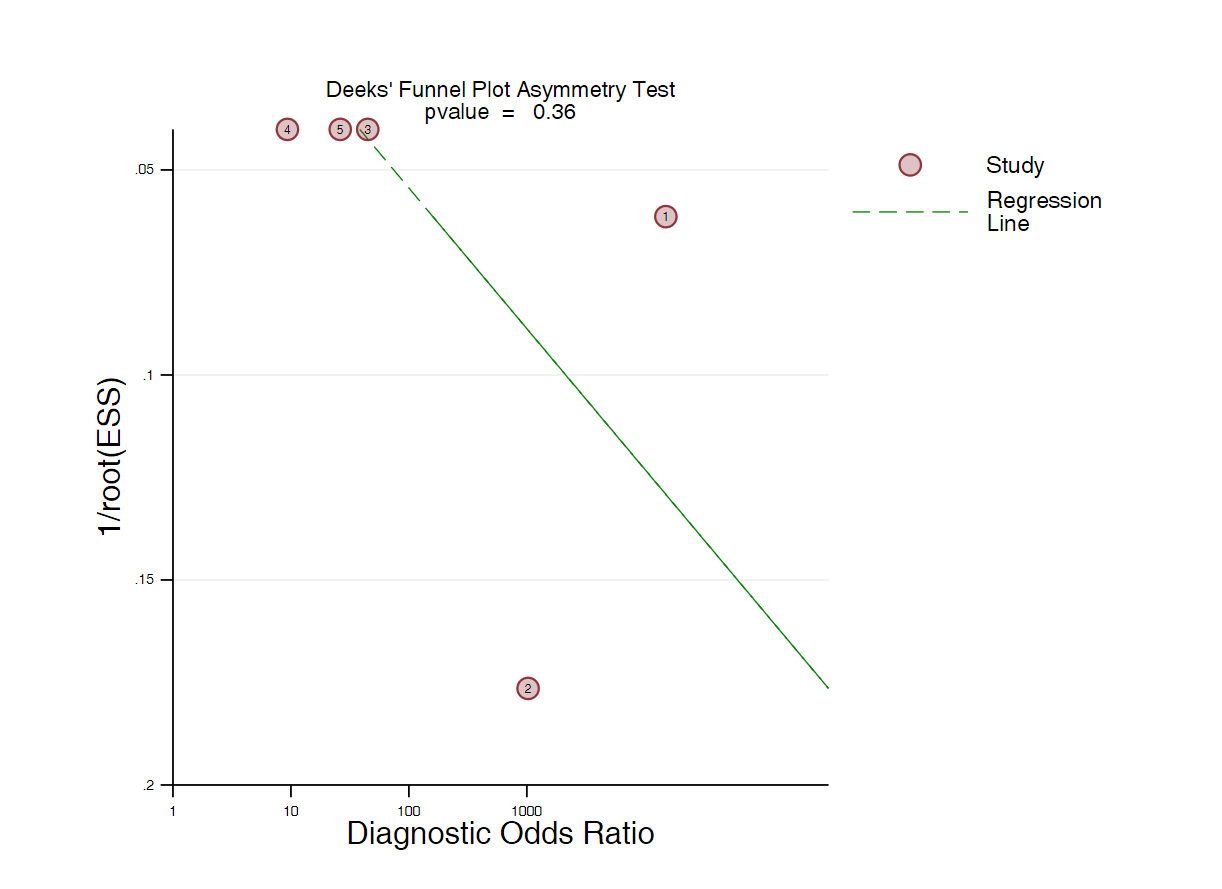


**Supplementary Figure 13.** Publication bias using proteinopathy PET imaging (AD vs. HC)

Funnel plots suggested there was a publication bias.


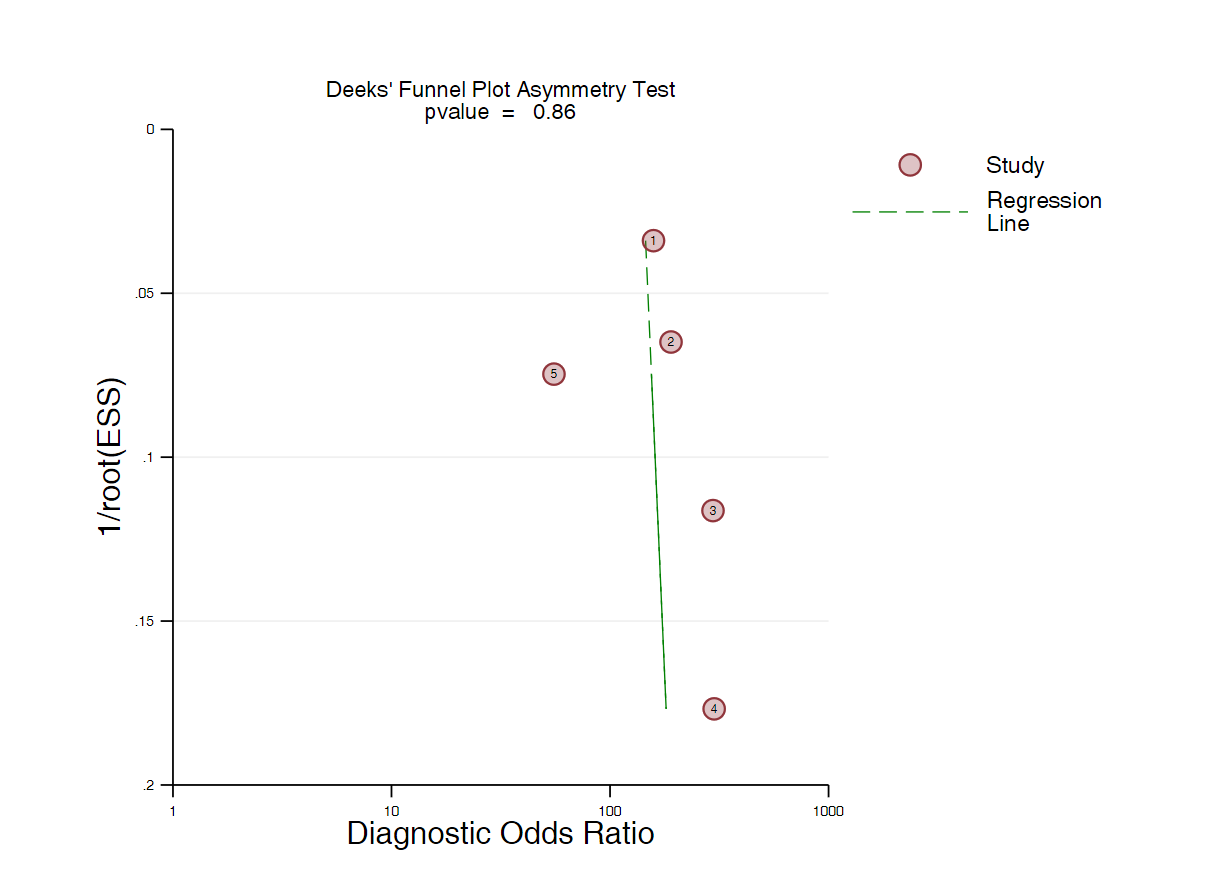


**Supplementary Figure 14.** Publication bias using ^18^F-FDG imaging (AD vs. HC)

Funnel plots suggested there was a publication bias.


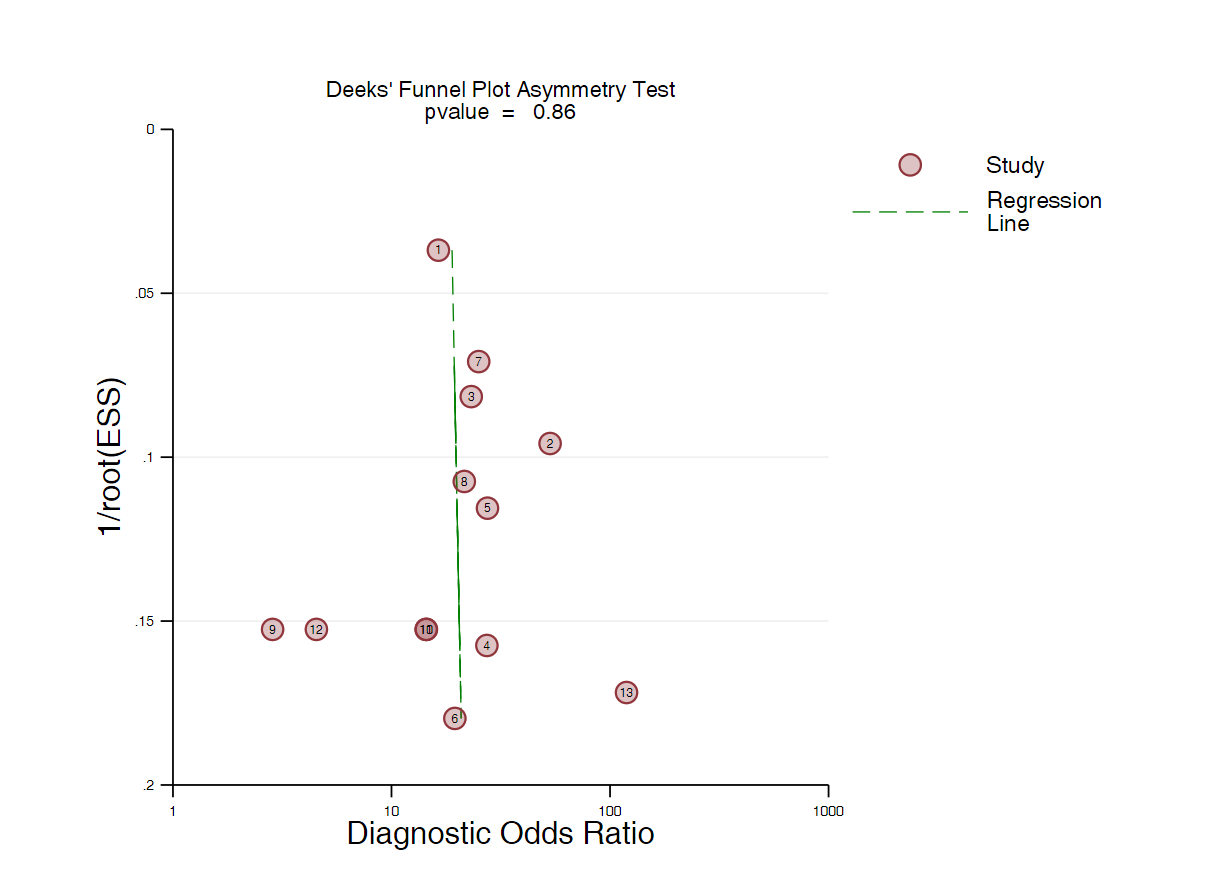


**Supplementary Figure 15.** Publication bias using ^18^F-FDG imaging (AD vs. MCI)

Funnel plots suggested there was a publication bias.


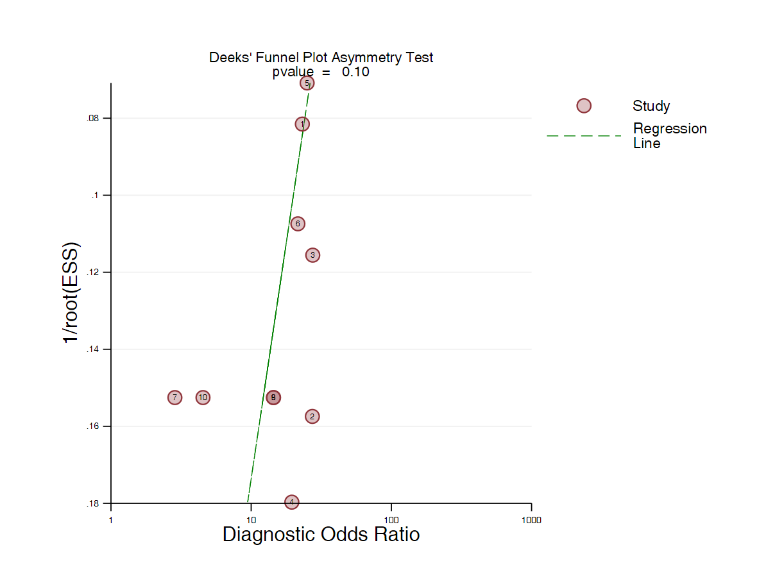

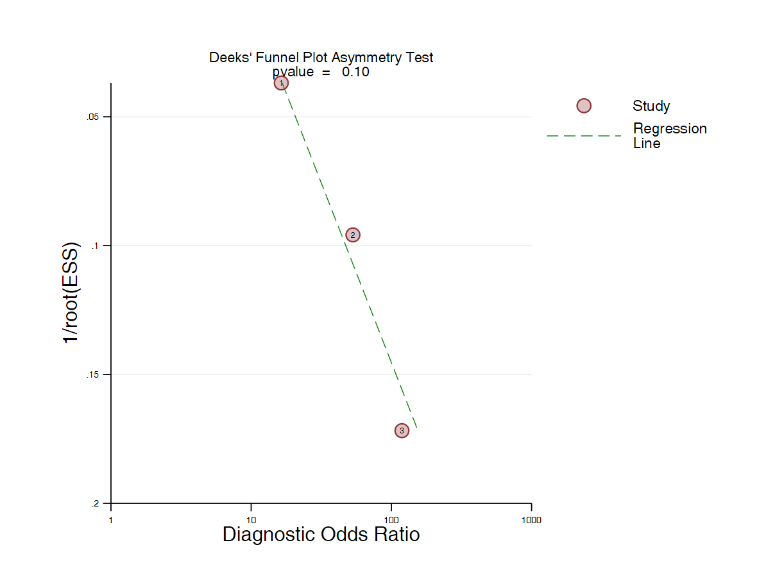


**Supplementary Figure 16.** Publication bias using DL- and ML- assisted ^18^F-FDG imaging (AD vs. MCI)

**Figure 16.1** DL (Deeks’ test showed no evidence of small-study effects (p =0.10)).

**Figure 16.2** ML (Deeks’ test showed no evidence of small-study effects (p = 0.10)).


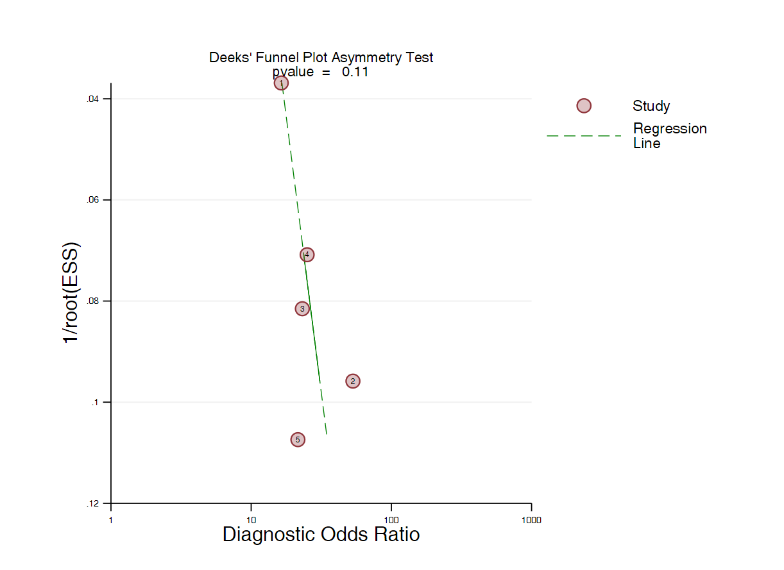

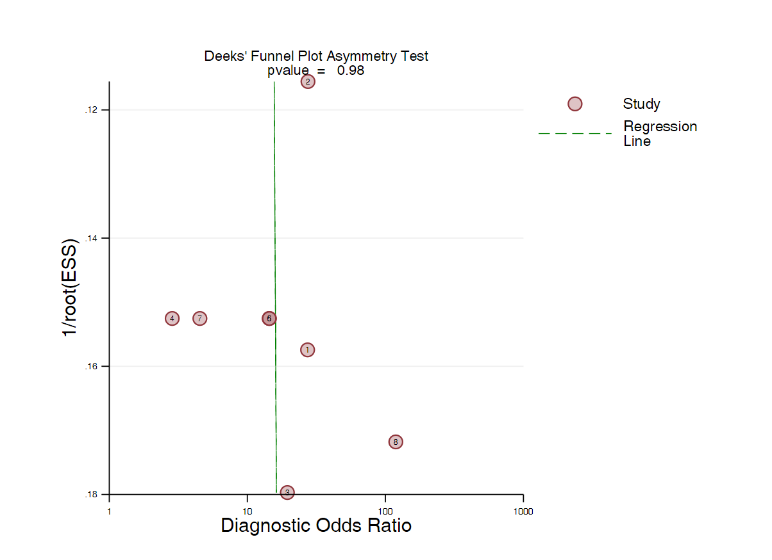
**Supplementary Figure 17.** Publication bias using ^18^F-FDG imaging based on sample size (AD vs. MCI)

**Figure 17.1** Sample size ≥ 100 (Deeks’ test showed no evidence of small-study effects (p = 0.11)).

**Figure 17.2** Sample size < 100 (Deeks’ test showed no evidence of small-study effects (p = 0.98)).


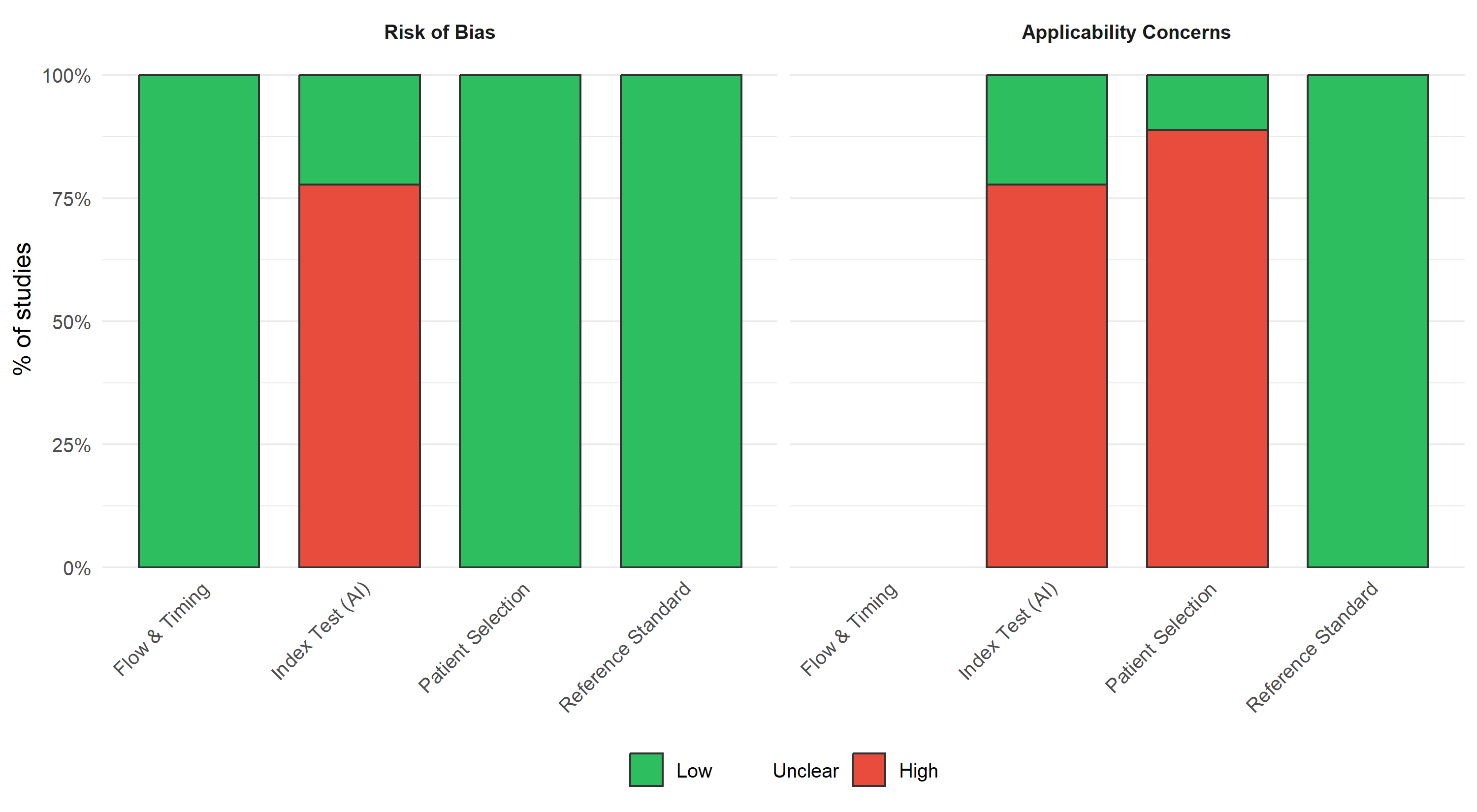


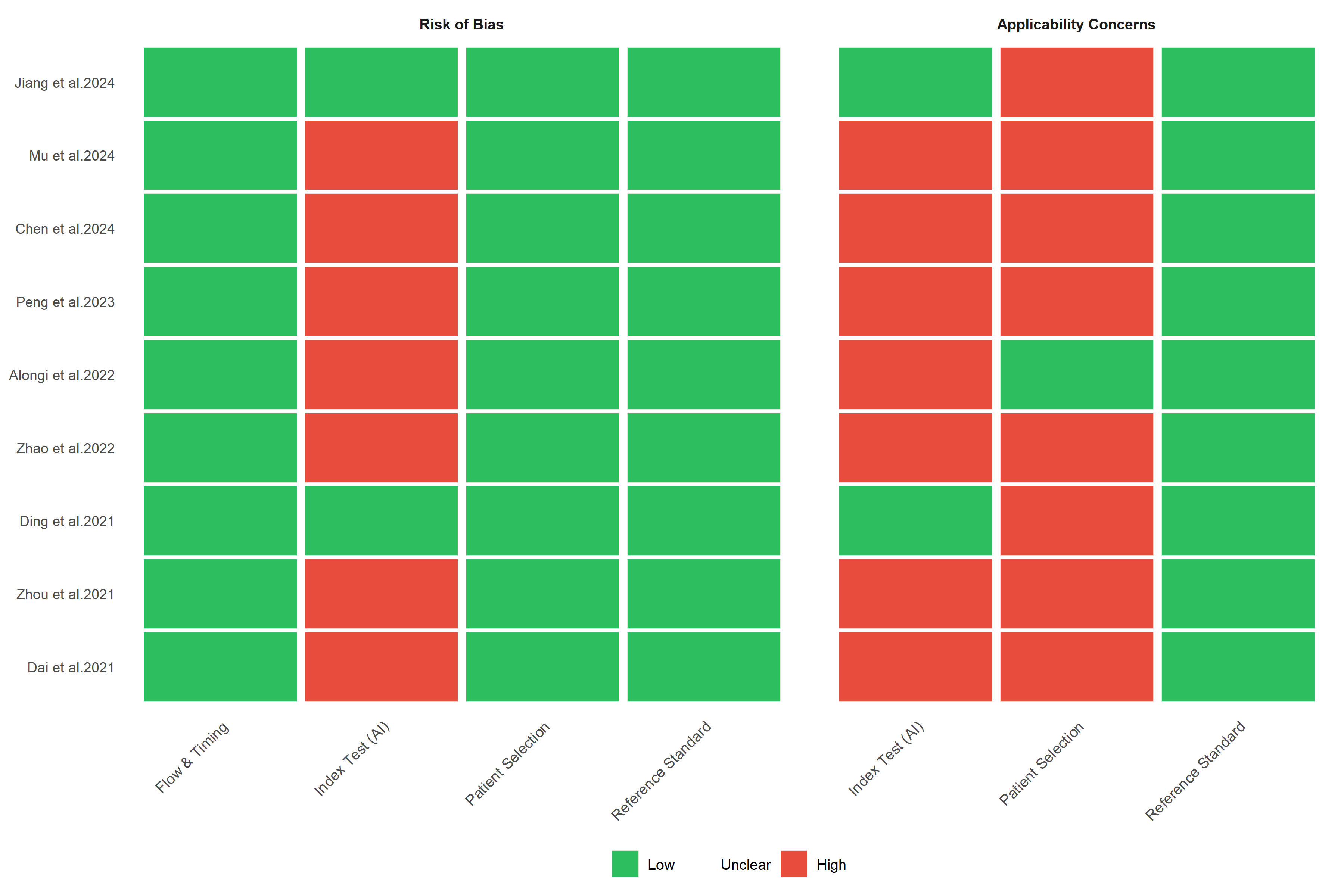


**Supplementary Figure 18.** QUADAS-AI summary plot and risk of bias and concern of applicability for each item

**Supplementary Methods 1.** Search terms and search strategy

We show the search strategy for 1. PubMed 2. Embase 3. Web of Science.

**PubMed**

| Search number | Query | Sort By | Filters | Search Details | Results | Time |
| --- | --- | --- | --- | --- | --- | --- |
| 10 | "Nervous System Diseases"[MeSH Terms] AND ("Artificial Intelligence"[MeSH Terms] OR "Machine Learning"[MeSH Terms] OR "Deep Learning"[MeSH Terms]) AND ("radiomics"[MeSH Terms]) AND ('alzheimer') NOT ("review") AND ((humans[Filter]) AND (english[Filter])) | Most Recent | English, Humans | ((("Nervous System Diseases"[MeSH Terms] AND ("Artificial Intelligence"[MeSH Terms] OR "Machine Learning"[MeSH Terms] OR "Deep Learning"[MeSH Terms]) AND "radiomics"[MeSH Terms] AND ("alzheime s"[All Fields] OR "alzheimer disease"[MeSH Terms] OR ("alzheimer"[All Fields] AND "disease"[All Fields]) OR "alzheimer disease"[All Fields] OR "alzheimer"[All Fields] OR "alzheimer s"[All Fields] OR "alzheimers"[All Fields] OR "alzheimers s"[All Fields])) NOT "review"[All Fields]) AND ("humans"[MeSH Terms] AND "english"[Language])) AND ((humans[Filter]) AND (english[Filter])) | 4 | 8:40:42 |
| 9 | "Nervous System Diseases"[MeSH Terms] AND ("Artificial Intelligence"[MeSH Terms] OR "Machine Learning"[MeSH Terms] OR "Deep Learning"[MeSH Terms]) AND ("radiomics"[MeSH Terms]) NOT ("review") | Most Recent | English, Humans | (("Nervous System Diseases"[MeSH Terms] AND ("Artificial Intelligence"[MeSH Terms] OR "Machine Learning"[MeSH Terms] OR "Deep Learning"[MeSH Terms]) AND "radiomics"[MeSH Terms]) NOT "review"[All Fields]) AND ((humans[Filter]) AND (english[Filter])) | 108 | 8:34:19 |
| 8 | "Nervous System Diseases"[MeSH Terms] AND ("Artificial Intelligence"[MeSH Terms] OR "Machine Learning"[MeSH Terms] OR "Deep Learning"[MeSH Terms]) AND ("radiomics"[MeSH Terms]) NOT ("review") | Most Recent | English | (("Nervous System Diseases"[MeSH Terms] AND ("Artificial Intelligence"[MeSH Terms] OR "Machine Learning"[MeSH Terms] OR "Deep Learning"[MeSH Terms]) AND "radiomics"[MeSH Terms]) NOT "review"[All Fields]) AND (english[Filter]) | 109 | 8:33:05 |
| 3 | "Nervous System Diseases"[MeSH Terms] AND ("Artificial Intelligence"[MeSH Terms] OR "Machine Learning"[MeSH Terms] OR "Deep Learning"[MeSH Terms]) AND ("radiomics"[MeSH Terms]) NOT ("review") | Most Recent | | ("Nervous System Diseases"[MeSH Terms] AND ("Artificial Intelligence"[MeSH Terms] OR "Machine Learning"[MeSH Terms] OR "Deep Learning"[MeSH Terms]) AND "radiomics"[MeSH Terms]) NOT "review"[All Fields] | 109 | 8:32:42 |
| 7 | "Nervous System Diseases"[MeSH Terms] AND ("Artificial Intelligence"[MeSH Terms] OR "Machine Learning"[MeSH Terms] OR "Deep Learning"[MeSH Terms]) AND ("radiomics"[MeSH Terms]) NOT ("review") | Most Recent | Meta-Analysis, Review, Systematic Review | (("Nervous System Diseases"[MeSH Terms] AND ("Artificial Intelligence"[MeSH Terms] OR "Machine Learning"[MeSH Terms] OR "Deep Learning"[MeSH Terms]) AND "radiomics"[MeSH Terms]) NOT "review"[All Fields]) AND (meta-analysis[Filter] OR review[Filter] OR systematicreview[Filter]) | 0 | 8:32:39 |
| 6 | "Nervous System Diseases"[MeSH Terms] AND ("Artificial Intelligence"[MeSH Terms] OR "Machine Learning"[MeSH Terms] OR "Deep Learning"[MeSH Terms]) AND ("radiomics"[MeSH Terms]) NOT ("review") | Most Recent | Meta-Analysis, Review | (("Nervous System Diseases"[MeSH Terms] AND ("Artificial Intelligence"[MeSH Terms] OR "Machine Learning"[MeSH Terms] OR "Deep Learning"[MeSH Terms]) AND "radiomics"[MeSH Terms]) NOT "review"[All Fields]) AND (meta-analysis[Filter] OR review[Filter]) | 0 | 8:32:36 |
| 5 | "Nervous System Diseases"[MeSH Terms] AND ("Artificial Intelligence"[MeSH Terms] OR "Machine Learning"[MeSH Terms] OR "Deep Learning"[MeSH Terms]) AND ("radiomics"[MeSH Terms]) NOT ("review") | Most Recent | Meta-Analysis | (("Nervous System Diseases"[MeSH Terms] AND ("Artificial Intelligence"[MeSH Terms] OR "Machine Learning"[MeSH Terms] OR "Deep Learning"[MeSH Terms]) AND "radiomics"[MeSH Terms]) NOT "review"[All Fields]) AND (meta-analysis[Filter]) | 0 | 8:32:33 |
| 4 | ("Nervous System Diseases"[MeSH]) AND ("Artificial Intelligence"[MeSH] OR "Machine Learning"[MeSH] OR "Deep Learning"[MeSH]) AND ('radiomics') | Most Recent | Meta-Analysis | ("Nervous System Diseases"[MeSH Terms] AND ("Artificial Intelligence"[MeSH Terms] OR "Machine Learning"[MeSH Terms] OR "Deep Learning"[MeSH Terms]) AND ("radiomics"[MeSH Terms] OR "radiomics"[All Fields] OR "radiomic"[All Fields])) AND (meta-analysis[Filter]) | 22 | 8:29:38 |
| 2 | ("Nervous System Diseases"[MeSH]) AND ("Artificial Intelligence"[MeSH] OR "Machine Learning"[MeSH] OR "Deep Learning"[MeSH]) AND ('radiomics') | Most Recent | | "Nervous System Diseases"[MeSH Terms] AND ("Artificial Intelligence"[MeSH Terms] OR "Machine Learning"[MeSH Terms] OR "Deep Learning"[MeSH Terms]) AND ("radiomics"[MeSH Terms] OR "radiomics"[All Fields] OR "radiomic"[All Fields]) | 534 | 8:29:31 |
| 1 | ("Nervous System Diseases"[MeSH]) AND ("Positron-Emission Tomography"[MeSH] OR "Tomography, Emission-Computed, Single-Photon"[MeSH]) AND ("Artificial Intelligence"[MeSH] OR "Machine Learning"[MeSH] OR "Deep Learning"[MeSH]) AND ("Image Processing, Computer-Assisted"[MeSH] OR "Pattern Recognition, Automated"[MeSH]) - Saved search | Most Recent | from 2016 - 2025 | ("Nervous System Diseases"[MeSH Terms] AND ("Positron-Emission Tomography"[MeSH Terms] OR "tomography, emission computed, single photon"[MeSH Terms]) AND ("Artificial Intelligence"[MeSH Terms] OR "Machine Learning"[MeSH Terms] OR "Deep Learning"[MeSH Terms]) AND ("image processing, computer assisted"[MeSH Terms] OR "pattern recognition, automated"[MeSH Terms])) AND (2016:2025[pdat]) | 71 | 8:23:57 |

**Embase**

| Embase session results (11 Apr 2025) | | |
| --- | --- | --- |
| No. | Query | Results |
| #7 | ('neurologic disease'/exp OR 'neurologic disease') AND ('radiomics'/exp OR 'radiomics') AND ('artificial intelligence'/exp OR 'artificial intelligence') NOT ('literature' OR 'alzheimer disease') AND [2016-2025]/py | 467 |
| #6 | ('neurologic disease'/exp OR 'neurologic disease') AND ('radiomics'/exp OR 'radiomics') AND ('artificial intelligence'/exp OR 'artificial intelligence') NOT 'alzheimer disease' AND [2016-2025]/py | 540 |
| #5 | #4 AND 'alzheimer disease'/dm | 35 |
| #4 | ('neurologic disease'/exp OR 'neurologic disease') AND ('radiomics'/exp OR 'radiomics') AND ('artificial intelligence'/exp OR 'artificial intelligence') NOT 'literature' AND [2016-2025]/py | 502 |
| #3 | #1 AND 'review'/it AND 'meta analysis' | 20 |
| #2 | #1 AND 'Review'/it | 174 |
| #1 | ('neurologic disease'/exp OR 'neurologic disease') AND ('radiomics'/exp OR 'radiomics') AND ('artificial intelligence'/exp OR 'artificial intelligence') AND [2016-2025]/py | 579 |

**Web of Science**

| Entitlements | Type | Search Query | Database | Results | Date Run |
| --- | --- | --- | --- | --- | --- |
| - WOS: 1985 to 2025 - KJD: 1980 to 2025 - MEDLINE: 1950 to 2025 - PPRN: 1991 to 2025 - PQDT: 1637 to 2025 - SCIELO: 2002 to 2025 | Search | TS=(  (Neurologic OR Neurology OR "brain disease" OR "central nervous system"   OR stroke OR "multiple sclerosis" OR Parkinson* OR Alzheimer* OR epilepsy)  AND  (Radiomic* OR "imaging biomarker*" OR "quantitative imaging"   OR "texture analysis")  AND  (AI OR "artificial intelligence" OR "machine learning" OR "deep learning"   OR "neural network*" OR "computer-assisted diagnosis") ) and Preprint Citation Index (Exclude – Database) and Article (Document Types) and Article (Document Types) and Neurosciences Neurology or Radiology Nuclear Medicine Medical Imaging (Research Areas) and 2025 or 2024 or 2023 or 2022 or 2021 or 2020 (Publication Years) and Review Article (Exclude – Document Types) and Article (Document Types) and Article (Document Types) and Alzheimer (Search within topic) | All Databases | 124 | Fri Apr 11 2025 20:47:57 GMT+0800 (GMT+08:00) |

**Supplementary Methods 2.** Inclusion/exclusion criteria of literature

| PICOS | Inclusion | Exclusion |
| --- | --- | --- |
| P | individuals with a confirmed diagnosis of AD, MCI and healthy controls(individuals with no significant clinical symptoms related to AD) | Participants with no relation to Alzheimer disease and not included in a concerning study as HC. No restrictions will be applied regarding age, sex, or ethnicity. |
| I | neurological diagnosis made based on positron emission tomography radiomics with AI method | Using other medical imaging approaches but not PET imaging was excluded (e.g., SPECT, MRI/fMRI). |
| C | Comparator: conventional PET visual read or SUVR threshold. Reference standard: histopathology or ≥12-month follow-up with clinical consensus diagnosis. | Studies that did not differentiate between AI-assisted PET imaging and clinical diagnosis were deemed ineligible. Studies that did not involve fully automated AI or a classification task. |
| O | Primary: sensitivity, specificity, AUC (AD vs HC; AD vs MCI). Secondary: LR+, LR−, DOR, threshold effects, calibration/heterogeneity. | Relevant outcomes were missing. |
| S | The present analysis includes diagnostic accuracy studies (cross-sectional or cohort designs with an index test and a reference standard). | 1) Articles that have not undergone the peer-review process or been published. 2) Quasi-experimental studies and crossover studies. |
